# Explainable Machine Learning for Punching Shear and Post-Failure Integrity

**DOI:** 10.3390/ma19204351

**Published:** 2026-10-16

**Authors:** Ahmed N. Elbelacy, Hassan Mohamed Ibrahim, Mina A. Mikaeel, Andrew Gouda

**Affiliations:** 1 Construction and Building Engineering Department, Arab Academy for Science, Technology and Maritime Transport, Port Said 42511, Egypt; andrew.gouda@aast.edu; 2 Civil Engineering Department, Faculty of Engineering, Port Said University, Port Said 42526, Egypt; hassanghattass@gmail.com; 3 KS Engineers, P.C., Newark, NJ 07102, USA; mmikaeel@kseng.com

**Keywords:** punching shear, slab–column connections, machine learning, source-grouped validation, explainable artificial intelligence, conformal prediction, post-failure integrity

## Abstract

Punching shear is a brittle failure mode in reinforced-concrete slab–column connections, requiring predictive models with explicit validity limits. This study develops a mechanics-informed machine-learning workflow using 1068 quality-controlled specimens from 111 literature sources. XGBoost achieved R^2^ = 0.932 under a specimen-random five-fold benchmark, while leakage-safe nested source-grouped validation yielded R^2^ = 0.858 and a fully source-disjoint holdout yielded R^2^ = 0.793. Performance was substantially weaker for scarce edge and corner connections than for interior connections, demonstrating configuration-dependent transfer limitations. Predictive-importance analyses identified geometry and loading as the dominant variable blocks; these associations were not causal. Conformal prediction provided marginal predictive-uncertainty diagnostics, including 97.90% coverage with a 507.14 kN mean interval width on the source-disjoint test, but does not establish configuration-conditional coverage or structural reliability. Bounded perturbations were likewise interpreted as sensitivity analysis rather than reliability assessment. A separate nine-specimen geogrid program provided complementary post-failure evidence; quantitative post-peak conclusions were restricted to the three specimens with complete records. The workflow supports validation-aware research screening within the represented domain while emphasizing source separation and configuration-specific applicability.

## 1. Introduction

Punching shear is a critical brittle failure mechanism in reinforced-concrete (RC) flat slabs and slab–column connections. Unlike flexural failure, punching may develop with limited deformation and relatively little warning, potentially compromising local load transfer and, in severe cases, contributing to progressive or disproportionate structural damage. Reliable assessment of punching shear resistance is therefore essential for the safe design and evaluation of flat-slab systems [1,2,3,4,5,6,7,8,9]. The response is governed by interacting geometric, material, reinforcement, loading, and connection-specific parameters, which complicates prediction across the range of configurations represented in experimental databases. The broader application of artificial intelligence in structural engineering and experimental studies of slab–column punching behavior further motivate data-driven assessment [10,11].

Machine-learning (ML) methods have consequently received increasing attention as complementary tools for punching-shear prediction. Related assessment and database studies have addressed progressive-collapse criteria and experimental or code-based datasets [12,13,14,15], while experimental work has also examined geogrid-reinforced slabs [16]. Several ML studies have reported strong predictive performance for the databases considered [12,17,18,19]. General interpretability research and numerical modeling of UHPFRC slabs provide complementary methodological and mechanics context [20,21], while further punching-shear ML applications are reported in [22,23]. The use of ML for punching-shear prediction is therefore already established and, by itself, is not the novelty pursued in the present study.

Recent punching-shear studies have extended this work beyond point prediction. Nguyen et al. [12] developed an XGBoost model for 497 interior slab–column connections, compared it with alternative ML models, design codes, and empirical formulations, examined relative input importance, and implemented a graphical interface. The complementary literature has examined internal GFRP/grating reinforcement and comparative shear-prediction models [24,25], automated ensemble learning and progressive-collapse response in reinforced-concrete systems [26,27], and the methodological foundations of SHAP, conformal prediction, geogrid-reinforced slab behavior, and structural reliability/probability analysis [28,29,30,31,32,33]. Mangalathu et al. [34] evaluated eight regression approaches using 380 two-way RC flat-slab tests at interior supports without transverse reinforcement, selected XGBoost following random training-testing evaluation, compared its predictions with established design provisions and empirical models, and used SHapley Additive exPlanations (SHAP) for model interpretation. Liang et al. [17] used XGBoost together with SHAP and partial-dependence analysis to examine punching resistance, failure behavior, and influential structural parameters. Shen et al. [35] coupled machine-learning surrogate modeling based on 610 experimental records with Monte Carlo simulation and reliability-oriented assessment. Yan and Xie [22] combined Bayesian optimization, multiple ML algorithms, SHAP analysis, design provisions, and Critical Shear Crack Theory (CSCT) for 337 flat slabs with transverse reinforcement. More recently, Ren and Liu [36] introduced probabilistic NGBoost modeling, SHAP and Sobol sensitivity analyses, and Monte Carlo-based reliability assessment using 594 experimental tests, while Shah et al. [37] compared eight ML algorithms using 378 validated flat-slab tests and employed SHAP and partial-dependence plots (PDPs) for explainability-oriented interpretation. Collectively, these studies demonstrate that high predictive accuracy, explainable artificial intelligence (XAI), optimization, probabilistic modeling, reliability-oriented analysis, code benchmarking, and practical implementation have already become important components of punching-shear ML research.

A more fundamental issue concerns how predictive performance is validated when experimental data are compiled from multiple literature sources. Specimens originating from the same experimental campaign may share materials, geometric families, reinforcement details, loading arrangements, test procedures, instrumentation, and laboratory-specific practices. Consequently, a random specimen-level split can place closely related observations from the same experimental source in both model-development and evaluation subsets. Under such a protocol, strong test performance may reflect a combination of interpolation within represented experimental programs and transfer across programs rather than cross-study transfer alone.

This distinction becomes important when the intended inference extends beyond individual withheld specimens. Source-grouped cross-validation provides a stricter evaluation by keeping records associated with the same literature source within a common partition, thereby preventing specimens from that source from being divided between training and validation folds. A fully source-disjoint holdout imposes a further test by evaluating the model on experimental sources excluded entirely from model development. Comparing specimen-random, source-grouped, and source-disjoint protocols therefore provides a direct means of determining how estimated predictive performance changes as the separation between model-development and evaluation evidence becomes progressively stricter.

Aggregate performance metrics can additionally conceal configuration-dependent limitations. Literature-derived punching-shear databases are frequently dominated by interior slab–column connections, whereas edge and corner configurations are substantially less represented. A strong global coefficient of determination or low overall prediction error may therefore be driven primarily by performance for the dominant structural configuration. Connection-specific evaluation is consequently needed to determine whether cross-study predictive performance remains consistent across interior, edge, and corner connections or deteriorates where experimental support is sparse.

Interpretability should likewise be considered within this validation context. SHAP and related feature-attribution methods can identify variables associated with model predictions, but they do not establish causal structural mechanisms, particularly when measured variables and engineered descriptors are correlated. Their engineering value is strengthened when feature attribution is complemented by independent diagnostics such as held-out grouped permutation importance and feature ablation and when the resulting patterns are examined against established punching-shear mechanics. The relevant question is therefore not simply whether an ML model can be explained, but whether model-derived predictive associations remain useful and physically plausible under source-separated evaluation.

Predictive uncertainty and empirical support constitute a related limitation. Point predictions alone do not indicate the magnitude of plausible prediction error or whether a queried case is adequately represented by the available predictor space. Conformal prediction can provide marginal prediction intervals under its stated exchangeability conditions, while applicability-domain screening can identify cases with weak empirical support. These diagnostics are distinct from formal structural reliability analysis and do not replace code-based safety verification. Instead, they provide complementary information regarding predictive uncertainty and data support, particularly when transfer to experimental sources excluded from model development is examined.

Table 1 summarizes the methodological positioning of representative punching-shear ML studies relevant to the present work. The literature has already demonstrated ensemble learning, explainability, optimization, probabilistic and reliability-oriented analysis, code benchmarking, and practical implementation [12,17,22,34,35,36,37]. The computational components used in this study draw on established Random Forest, XGBoost, CatBoost, SHAP, and conformal-prediction formulations [38,39,40,41,42], while specialized punching-shear ML applications have also addressed lightweight, fiber-reinforced, FRP-reinforced, and strengthened slab systems [43,44,45,46,47]. Against this background, the present study focuses on a validation issue that remains insufficiently characterized in the punching-shear ML literature: how estimated predictive performance changes when evaluation is progressively tightened from specimen-random testing to source-grouped cross-validation and fully source-disjoint testing, and whether the resulting cross-study transferability remains consistent across connection configurations. Predictive uncertainty and applicability-domain diagnostics are then examined within this transfer-oriented framework to identify where empirical support or predictive precision becomes limiting.

SHAP: SHapley Additive exPlanations; PDP: partial-dependence plot; CSCT: Critical Shear Crack Theory; GUI: graphical user interface. “Not identified in the reported evaluation” is used conservatively where a dedicated source-grouped, source-disjoint, or configuration-transfer assessment was not identified in the methodological evaluation considered here; it should not be interpreted as a claim that the broader study contains no related analysis. Source-grouped validation denotes evaluation in which records from the same literature source remain within the same partition, whereas fully source-disjoint testing denotes evaluation on literature sources excluded entirely from model development. The comparison is intended to distinguish validation and transferability evidence rather than to rank studies according to predictive accuracy.

Against this background, the central research question of the present study is:

To what extent does predictive performance persist when punching-shear ML models are evaluated under progressively stricter separation of experimental sources, from specimen-level evaluation to cross-study transfer?

This question is addressed through four specific research questions:

RQ1—Validation-induced generalization gap: How does estimated punching-shear predictive performance change when evaluation progresses from specimen-random validation to source-grouped cross-validation and fully source-disjoint testing?

RQ2—Configuration-dependent transferability: Is cross-study predictive performance consistent across interior, edge, and corner slab–column connections, or can aggregate accuracy conceal configuration-specific generalization limitations?

RQ3—Mechanics-informed transferability: Do mechanics-informed descriptors and model-derived importance patterns remain useful and physically plausible under source-separated evaluation?

RQ4—Uncertainty and applicability under transfer: Can predictive uncertainty and empirical support be characterized under source-separated transfer, and where do interval width, applicability-domain limitations, and sparse structural configurations constrain engineering interpretation?

To answer these questions, a quality-controlled database of 1068 experimentally tested slab–column connections from 111 traceable literature-source groups is examined under a hierarchical validation strategy comprising specimen-random evaluation, source-grouped cross-validation, and a fully source-disjoint holdout. Random Forest, XGBoost, and CatBoost are first compared under the reproduced specimen-level benchmark, after which XGBoost is subjected to source-aware validation and diagnostic analyses. Mechanics-informed feature engineering is complemented by SHAP, held-out grouped permutation importance, and feature ablation. Predictive limitations are further examined using marginal split-conformal diagnostics, applicability-domain screening, connection-specific performance assessment, and bounded input-perturbation analysis. Nominal ACI 318-19 and Eurocode 2 predictions are retained only as engineering error benchmarks and not as targets for replacement.

A separate nine-specimen experimental program using steel- and geogrid-reinforced cementitious slabs provides complementary post-failure mechanics observations. Because complete post-peak records are available for only three specimens, quantitative post-failure conclusions are restricted to those records and are not presented as external validation of the ML predictor or as evidence of universal geogrid superiority. The experimental program therefore serves as complementary mechanics evidence rather than as validation of predictive generalization.

Finally, the evaluated workflow is implemented in a research-screening interface that reports the point prediction together with applicability-domain status, reference uncertainty information, and relevant safeguards. The interface is intended as an operational translation of the study findings rather than as an independent methodological novelty, a validated structural-design tool, or a substitute for code-based verification and engineering review.

The contribution of the present study therefore lies in a validation-centered assessment of punching-shear machine learning. Rather than treating high specimen-level accuracy as sufficient evidence of generalizability, the study quantifies how predictive performance changes as experimental-source separation becomes progressively more stringent, examines whether the resulting transfer limitations are configuration dependent, and relates those limitations to mechanics-informed interpretation, predictive uncertainty, and applicability-domain support. This framing is intended to define both the predictive capability and the evidential limits of data-driven punching-shear assessment within the represented literature domain.

## 2. Database Development and Feature Preparation

**Figure 1:**
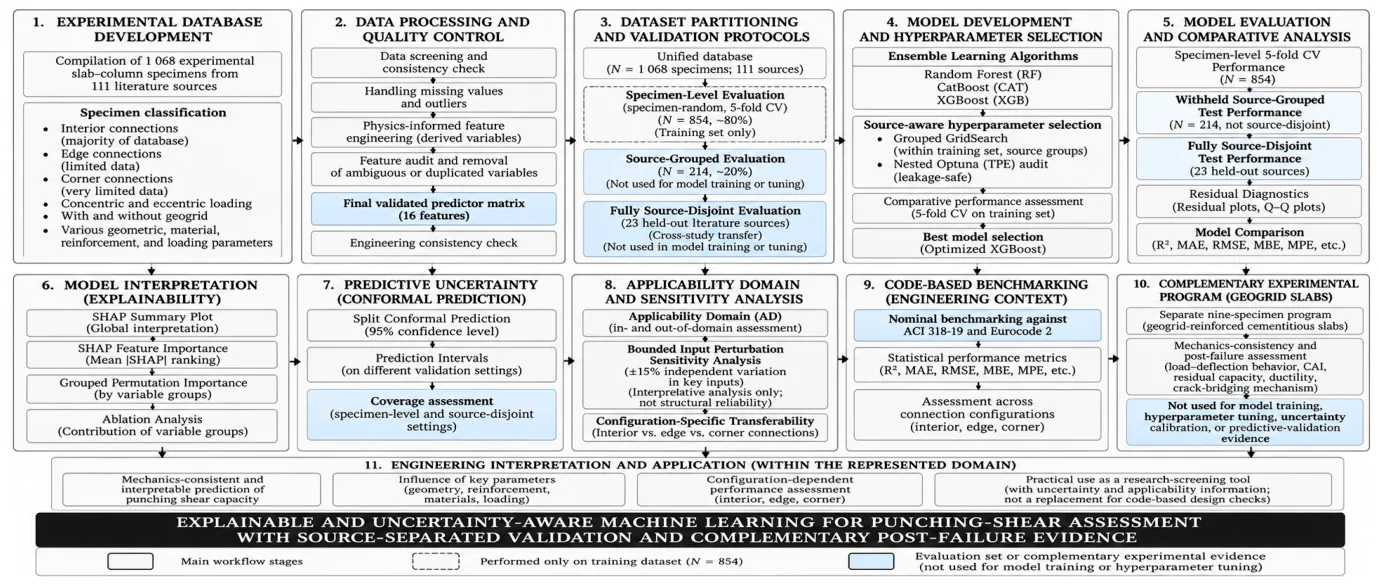
Overall workflow of the proposed explainable and uncertainty-aware machine-learning framework for punching-shear assessment, including specimen-level evaluation, source-separated validation, predictive-uncertainty and applicability analyses, and complementary post-failure experimental evidence.

Figure 1 summarizes the study workflow, comprising database assembly and quality control, mechanics-informed feature construction, specimen-level and source-separated validation, model interpretation, predictive-uncertainty and applicability-domain assessment, code benchmarking, and a separate post-failure mechanics investigation.

The verified database was initially partitioned at specimen level into 854 training and 214 withheld test specimens using random_state = 42. Because literature-source identifiers were not used as grouping constraints in this benchmark, specimens from the same experimental campaign may occur in both partitions; these results therefore characterize within-database interpolation rather than fully independent cross-study transfer. Cross-study generalization was subsequently examined using source-grouped cross-validation and a fully source-disjoint holdout.

SHAP, conformal prediction, applicability-domain screening, and bounded perturbation analysis were used as complementary diagnostic tools. The separate geogrid experiments were reserved for mechanics-consistency and post-failure assessment and were not used as predictive-validation evidence.

### 2.1. General Description of the Experimental Database

The final quality-controlled database contains 1068 published slab–column tests with measured punching capacity, Vexp, as the prediction target. Records were harmonized in consistent units and screened for missing, nonphysical, duplicate, and traceability-related inconsistencies.

The database contains 983 interior (92.0%), 51 edge (4.8%), and 34 corner (3.2%) connections under concentric and eccentric loading. This imbalance is explicitly considered in the subsequent configuration-specific transfer assessment and limits inference for the sparse edge and corner subsets.

The experimentally measured punching shear capacity, Vexp, was the target. The database was divided at specimen level into 854 training records (80%) and 214 withheld test records (20%) using random_state = 42. The withheld set was not used for fitting or tuning, but it was not isolated by publication campaign. Accordingly, it is termed a withheld specimen-level test set rather than an independent or external holdout.

**Figure 2:**
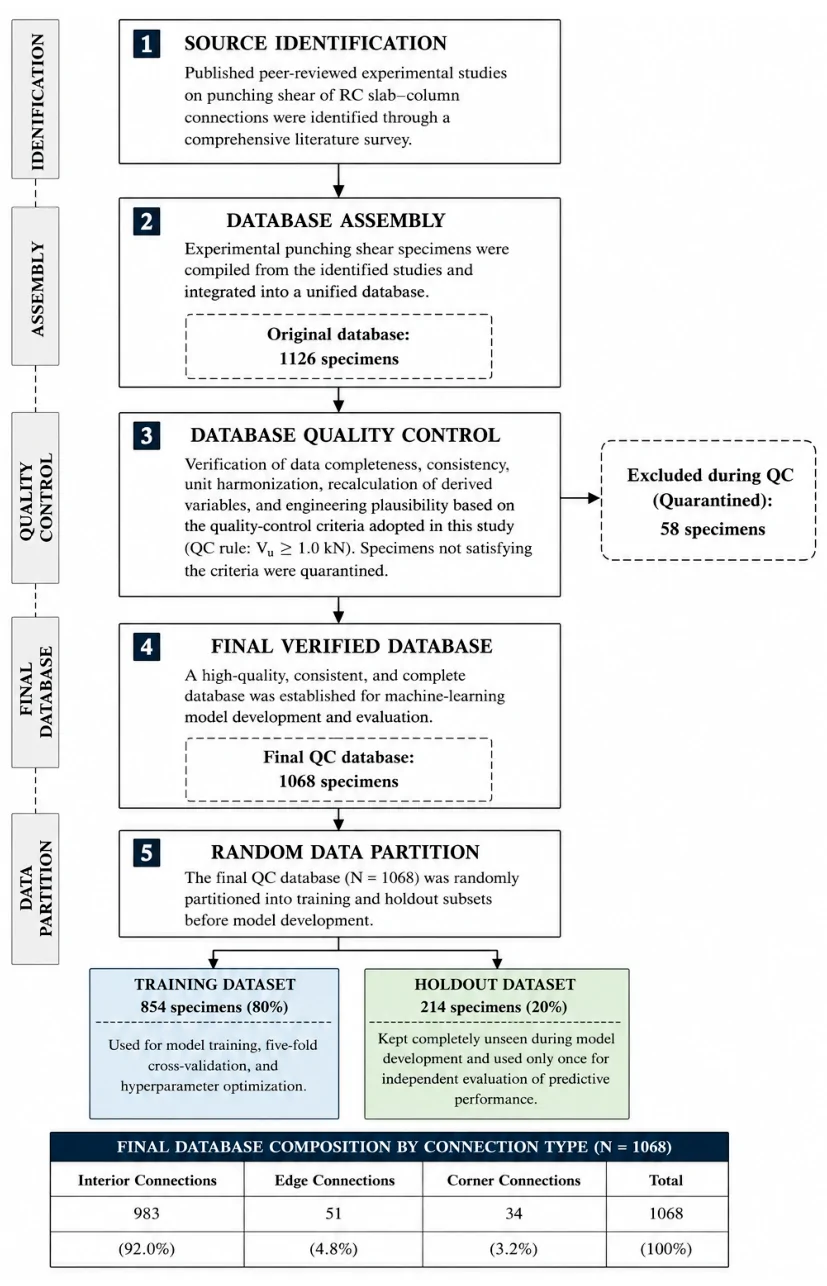
Database compilation, quality control, and data-partitioning procedure adopted for assembling the unified experimental database used for machine-learning model development and validation.

Figure 2 summarizes the database compilation, quality-control procedure, feature preparation, and subsequent dataset partitioning adopted prior to machine-learning model development.

### 2.2. Database Characterization, Traceability, and Scope

Table 2 summarizes the principal ranges and dispersion of the experimental variables. The database spans substantial variation in material, geometric, and resistance-related quantities; however, broad marginal ranges should not be interpreted as evidence of balanced or representative coverage, particularly given the strong predominance of interior connections.

**Figure 3:**
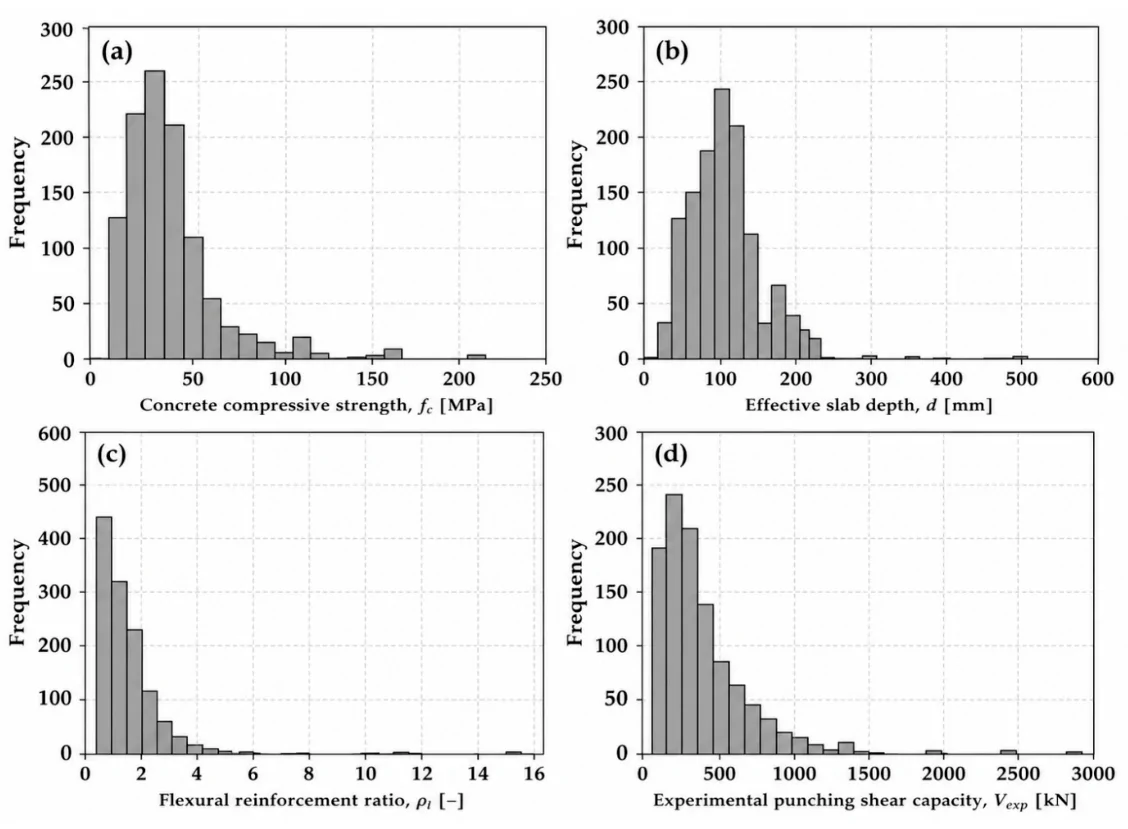
Distribution of the principal variables included in the assembled database: (**a**) concrete compressive strength (*f_c_*), (**b**) effective slab depth (*d*), (**c**) flexural reinforcement ratio (*ρ_l_*), and (**d**) experimental punching shear capacity (*V*_exp_).

Figure 3 shows the marginal distributions of concrete strength, slab depth, longitudinal reinforcement ratio, and capacity. The database stores *ρl* in percentage points (e.g., 1.00 denotes 1.00%). The maximum recorded value, 15.6%, corresponds to the record labeled “Hanai and Holanda.” Because this value could not be independently reconciled with a source worksheet in the available archive, it was retained for traceability and treated as an extreme record rather than evidence of representative coverage.

Table 3 summarizes the distribution of slab–column connection types. Interior connections constitute 983 specimens (92.0%), whereas edge and corner connections account for 51 (4.8%) and 34 (3.2%) specimens, respectively. This distribution reflects the predominance of interior slab–column connections in the published experimental literature rather than a deliberate sampling strategy.

The strong configuration imbalance means that results are most defensible for interior specimens; edge and corner predictions require cautious interpretation and further independent experimental evidence.

### 2.3. Input and Output Variables

The validated grouped analyses used 16 predictors: seven measured variables (*d*, *u*1, *fc*, *fy*, *ρl*, *Vf*, and *Ac*), eccentricity ratio *e*/*d*, three connection-type indicators, and five derived mechanics descriptors (√*fc*, *Ac*/*d*2, *ρlfy*, *ρl*√*fc*, and u1d). All 16 fields were complete in the 1068-record quality-controlled dataset. Column dimensions *c*1 and *c*2 and slab thickness *h* remain traceability fields in the source dataset but were not used in the validated 16-feature model; *h* is missing in 946 records.

The experimentally measured ultimate punching shear capacity *V*_exp_ was adopted as the output variable throughout this investigation. Table 4 summarizes the input and output variables used for machine-learning model development.

### 2.4. Feature Audit and Exclusion of Ambiguous Variables

Predictor selection balanced structural relevance with cross-study reporting. Effective depth and critical perimeter describe the shear-transfer region; concrete and reinforcement variables describe material resistance, crack control, dowel action, and redistribution; loaded area and e/d describe column geometry and eccentric loading; connection indicators distinguish interior, edge, and corner configurations. Derived descriptors encode prespecified mechanics combinations and are tested by ablation. Potential variables were excluded when they were unavailable in most studies, inconsistently defined, insufficiently traceable, dependent on incompatible assumptions, excessively incomplete, or derived from the response and therefore at risk of leakage.

The processed field m was excluded because the supplied data dictionary and source-traceability materials did not provide a stable definition, unit, or reproducible derivation across studies. “Ambiguous” therefore refers to unverifiable semantic and dimensional provenance, not merely low predictive value. Manual review of source papers cannot recover variables that individual campaigns did not report or defined incompatibly; heterogeneous reporting is an inherent limitation of literature-derived databases. Table 5 summarizes the audit outcome.

Following removal of the ambiguous descriptor m, the remaining variables had defined engineering meaning and traceable units. Their predictive importance is evaluated as association within the fitted model; it does not establish that the model recovered unique or causal punching-shear mechanisms.

Accordingly, Table 4 defines the variables used in the validated model, whereas Table 5 is limited to the audit decision trail for retained, traceability-only, and excluded fields. Predictive importance is interpreted as association within the fitted model rather than as causal identification.

### 2.5. Data Preprocessing and Mathematical Representation

The final validated predictor matrix contained 16 features. Seven directly reported engineering variables were retained: effective depth *d*, critical punching perimeter *u*1, concrete compressive strength *fc*, reinforcement yield strength *fy*, longitudinal reinforcement ratio *ρl*, fiber volume fraction *Vf*, and loaded column area *Ac*. Loading eccentricity was represented by the dimensionless ratio *e*/*d*. Connection configuration was represented by three one-hot indicator variables for interior, edge, and corner connections.

Five prespecified mechanics-informed descriptors were subsequently generated from the retained predictors: √*fc*, *Ac*/*d*2, *ρlfy*, *ρl*√*fc*, and *u*1*d*. These engineered descriptors were calculated without using the experimental punching-capacity target.

The resulting supervised regression problem can be written as

*Vpred* = *f*(*X*),

where *X* contains the 16 predictors listed in Table 4, and *Vpred* is the predicted ultimate punching shear capacity. The experimentally measured capacity *Vexp* was used only as the response variable.

No numerical normalization was required for the primary RF, XGBoost, or CatBoost fitting because all three are tree-based learners. However, connection type was explicitly represented by the three binary indicator variables listed in Table 4. Standardization was used only within the applicability-domain analysis, where distances were computed in training-fold standardized feature space.

Column dimensions *c*1 and *c*2 and total slab thickness *h* remain source-level traceability fields but were not used in the validated 16-feature model. In particular, *h* is missing in 946 of the 1068 records.

### 2.6. Physical Relevance of the Selected Variables

The selected predictors represent complementary aspects of punching-shear resistance and load transfer. Effective depth *d* and critical perimeter *u*1 characterize the shear-resisting geometry; *fc*, *ρl*, and *fy* describe concrete and reinforcement-related resistance; *Ac*, *e*/*d*, and connection type characterize loading and boundary geometry; and *Vf* represents fiber content where applicable. Engineered terms combine these measured quantities to represent coupled mechanics-informed descriptors and are interpreted as predictive associations rather than independent causal mechanisms.

### 2.7. Independent Mechanics-Consistency Experiment Using Geogrid-Reinforced Slabs

A separate nine-specimen experimental program examined steel- and uniaxial-geogrid-reinforced cementitious slabs. These specimens were not used for model training, tuning, uncertainty calibration, or predictive validation. Their role was restricted to complementary mechanics-consistency and post-punching integrity assessment, with quantitative post-peak comparisons limited to specimens for which complete digitized load–displacement records were available.

## 3. Machine Learning Framework and Model Development

### 3.1. Overview of the Proposed Framework

The modeling framework compared RF, XGBoost, and CatBoost using identical predictors and specimen-level partitions. The subsequent source-separated analyses focused on XGBoost to quantify cross-study transferability, while SHAP, uncertainty, applicability-domain, sensitivity, and engineering-benchmark analyses were applied as complementary diagnostics.

### 3.2. Problem Formulation

Punching capacity was formulated as a supervised nonlinear regression problem, *Vpred* = *f*(*X*), using the audited predictors defined in Table 4. Model selection and tuning were performed without access to the withheld evaluation data.

*f*:**X** → *V*_exp_

*Vpred* = *f*(*X*)

### 3.3. Data Preparation for Machine Learning

All model predictors were numerical. Scaling was unnecessary for RF, XGBoost, and CatBoost; standardization was applied only for distance-based applicability-domain assessment. Identical predictors and evaluation partitions were used for comparative model assessment.

### 3.4. Hyperparameter Optimization

GridSearchCV independently optimized each model on the training set using five-fold cross-validation. Selection prioritized mean R^2^, with MAE and RMSE used to check error magnitude and stability. Search spaces and reproducibility controls are reported in Table 6 and Table 7.

Table 6 and Table 7 document the searched hyperparameter ranges and the controls used in the reproducible fitting workflow.

### 3.5. Machine Learning Models

Random Forest (RF), XGBoost, and CatBoost were retained as established comparative tree-ensemble baselines for heterogeneous tabular regression [38,39,40]. RF provides a bagging-based ensemble, whereas XGBoost and CatBoost represent gradient-boosting alternatives with distinct training strategies. Their inclusion enables comparison across established nonlinear ensemble paradigms; none is presented as an algorithmic contribution of this study.

$$\hat{V}=\frac{1}{T}\sum_{t=1}^{T}f_{t}(x)$$

$$\sum_{i=1}^{n}l\left(V_{\exp,i},V_{\mathrm{pred},i}\right)+\sum_{k=1}^{K}\Omega (f_{k})$$

$$\hat{V}=\sum_{k=1}^{K}\alpha_{k}f_{k}(x)$$

### 3.6. Five-Fold Cross-Validation Strategy

Three progressively stricter evaluation levels were distinguished. First, five shuffled specimen-level folds (random_state = 42) reproduced conventional within-database validation without publication-level separation. Second, five-fold GroupKFold used the complete verbatim Reference field to keep each of the 111 literature sources entirely within either training or validation in every fold.

Third, a source-disjoint GroupShuffleSplit held out 23 complete sources after model selection using only the remaining 88 sources. These protocols respectively characterize specimen-level interpolation, transfer among represented studies, and held-out-study transfer.

### 3.7. Performance Evaluation Metrics

Predictive performance was quantified using R^2^, MAE, RMSE, MBE, MPE, and MAPE, where *Vexp*, *Vpred*, and *n* denote the experimental capacity, predicted capacity, and sample size, respectively. MAE and RMSE quantify absolute prediction error, whereas MBE, MPE, and MAPE characterize bias and relative error. The metrics were calculated as follows:

The coefficient of determination is defined as

$$R^{2}=1-\frac{\sum_{i=1}^{N}(V_{\exp,i}-V_{\mathrm{pred},i}{)}^{2}}{\sum_{i=1}^{N}(V_{\exp,i}-{\bar{V}}_{\exp}{)}^{2}}$$

The mean absolute error is expressed as

$$\mathrm{MAE}=\frac{1}{N}\sum_{i=1}^{N}|V_{\exp,i}-V_{\mathrm{pred},i}|$$

The root mean square error is given by

$$\mathrm{RMSE}=\sqrt{\frac{1}{N}\sum_{i=1}^{N}(V_{\exp,i}-V_{\mathrm{pred},i}{)}^{2}}$$

To quantify systematic prediction bias, the mean bias error is calculated as

$$\mathrm{MBE}=\frac{1}{N}\sum_{i=1}^{N}\left(V_{\exp,i}-V_{\mathrm{pred},i}\right)$$

The mean percentage error is defined as

$$\mathrm{MPE}=\frac{100}{N}\sum_{i=1}^{N}\left(\frac{V_{\exp,i}-V_{\mathrm{pred},i}}{V_{\exp,i}}\right)$$

The mean absolute percentage error is expressed as

$$\mathrm{MAPE}=\frac{100}{N}\sum_{i=1}^{N}|\frac{V_{\exp,i}-V_{\mathrm{pred},i}}{V_{\exp,i}}|$$

### 3.8. Holdout Validation Strategy

The reproduced specimen-level benchmark used the fixed 854/214 training/test partition (random_state = 42). The 214-specimen test set was excluded from fitting, hyperparameter selection, and conformal calibration. Because publication sources were not isolated across this split, its performance is reported explicitly as withheld specimen-level evaluation rather than external or source-independent validation. The optimized RF, XGBoost, and CatBoost models were evaluated on this set without further adjustment.

### 3.9. Source-Grouped Validation Analysis

Source identity was audited before grouped analysis. The nominal source column was empty, whereas Reference was complete for all 1068 records and contained 111 distinct verbatim labels; no labels were inferred or merged. Five-fold GroupKFold assigned every reference exclusively to training or validation in each fold, and the audited overlap was zero. XGBoost was selected using R^2^ over an explicitly reported 432-candidate grouped grid (2160 fits): n_estimators 300–800, max_depth 3–5, learning_rate 0.03–0.10, subsample and colsample_bytree 0.8–1.0, min_child_weight 1 or 3, and reg_lambda 1 or 3. The selected setting was n_estimators = 800, max_depth = 3, learning_rate = 0.03, subsample = 0.8, colsample_bytree = 1.0, min_child_weight = 1, reg_lambda = 1, and random_state = 42. This grouped grid is distinct from the larger legacy specimen-random search space in Table 6. Out-of-fold predictions supplied all grouped metrics. A separate 80/20 GroupShuffleSplit contained 88 training sources and 23 test sources; hyperparameters were returned by GroupKFold using training sources only. The retained hyperparameter settings and their reproducibility status are summarized in Table 7.

### 3.10. Applicability-Domain Assessment

For each source-grouped validation fold, predictors were standardized using the mean and standard deviation estimated from the training portion of that fold only. Applicability was then assessed in the standardized feature space using a five-nearest-neighbor (5-NN) distance criterion. For each training specimen, the mean Euclidean distance to its five nearest training neighbors was calculated using a self-excluded neighbor search. The fold-specific applicability-domain threshold was defined as the 95th percentile of the resulting training-sample mean 5-NN distance distribution. Each held-out specimen was subsequently standardized using the corresponding training-fold statistics, and its mean Euclidean distance to the five nearest training specimens was calculated. Specimens with distances not exceeding the fold-specific threshold were classified as inside the training-density envelope, whereas those exceeding the threshold were classified as outside the applicability domain. This procedure provides a local data-support diagnostic for identifying predictions made in sparsely represented regions of the predictor space; it is not a deterministic boundary of physical validity and does not convert model predictions into code-qualified design values.

### 3.11. Nested Bayesian Hyperparameter Optimization Audit

To test whether the coarse grouped grid constrained the conclusions, a leakage-safe nested Optuna/TPE audit was performed for XGBoost. Five outer GroupKFold folds supplied untouched source-separated evaluation data. Within each outer-training partition, five-fold GroupKFold minimized RMSE over 300 TPE trials, giving 1500 completed trials. All five outer and 25 inner train–validation source intersections were empty. The outer-validation records were not used for tuning. Early stopping was omitted because leakage-safe implementation would require another source-separated partition inside every inner-training fold.

The Bayesian search allowed 50–1500 estimators, depth 2–10, log-uniform learning rate 0.005–0.30, child weight 1–15, subsample and column fractions 0.50–1.00, gamma 0–1, log-uniform L1 penalty 10−8–10, and log-uniform L2 penalty 10−3–100. This audit evaluates sensitivity to a substantially wider search space; it does not redefine Optuna or GridSearch as the superior optimizer.

### 3.12. Physics-Informed Feature Engineering

Mechanics-informed descriptors were restricted to the five engineered inputs used in the validated 16-feature model: *u*1*d*, *ρlfy*, *ρl*√*fc*, *Ac*/*d*2, and √*fc*. These terms represent shear-resisting geometry, reinforcement capacity, reinforcement-concrete interaction, relative loaded-column geometry, and concrete-strength scaling, respectively. No additional *Vf*√*fc* interaction was used in the validated predictor matrix.

The engineered features were formulated only from measured predictor variables and are defined consistently with Table 4 and Table 5 as follows:

*F*1 = *u*1*d*;     *F*2 = *ρlfy*;     *F*3 = *ρl*√*fc*;     *F*4 = *Ac*/*d*2;     *F*5 = √*fc*

where *u*1*d* represents the effective shear-transfer region, *ρlfy* characterizes reinforcement capacity, *ρl*√*fc* represents a coupled reinforcement-concrete descriptor, *Ac*/*d*2 represents loaded-column geometry relative to effective depth, and √*fc* is the concrete-strength transformation. These definitions are the feature names used throughout the validated model and interpretation sections.

All engineered descriptors were calculated exclusively from predictor variables and independently of the experimental punching-capacity target. Accordingly, the feature-engineering step did not introduce target-derived information into the predictors. Source overlap and partition leakage were addressed separately through the grouped validation protocols.

The engineered descriptors compactly encode coupled mechanics-informed effects. Their incremental predictive contribution was modest under source-grouped ablation.

Furthermore, preserving the engineering meaning of the engineered descriptors facilitates mechanics-informed interpretation of the subsequent SHAP analysis. However, because original and engineered predictors are correlated, the resulting SHAP attributions are interpreted as model-specific predictive associations and mechanics-consistency evidence rather than as uniquely identifiable or causal physical contributions.

### 3.13. SHAP Explainability Analysis [41]

SHapley Additive exPlanations (SHAP) values were computed for the fitted XGBoost model to describe global and local predictive associations. Positive values raise a prediction relative to the baseline and negative values reduce it. Because original and engineered predictors are correlated, individual attribution is neither unique nor causal. A complementary held-out group permutation analysis aggregated variables into geometry/loading, longitudinal reinforcement, fibers, and concrete-strength blocks.

The SHAP prediction decomposition is expressed as

$$f(x)=\phi_{0}+\sum_{i=1}^{M}\phi_{i}$$

where *f*(*x*) is the predicted punching shear capacity, *ϕ*_0_ is the baseline prediction corresponding to the expected model output, *ϕ_i_* is the SHAP value representing the contribution of the *i*^th^ input variable, and *M* is the total number of input features.

Positive SHAP values indicate that an input variable increases the predicted punching shear capacity relative to the baseline prediction, whereas negative SHAP values reduce the predicted punching shear capacity.

SHAP summary and mean-absolute-importance plots were used to describe associations learned by the fitted model. Because *u*_1_, *d*, *u*_1_*d*, *ρ*_l_, *f*_γ_, *ρ*_l_*f*_γ_, *f_c_*, and *ρ*_l_√*f_c_* are correlated or algebraically related, individual attributions are not assumed to be unique or causally identifiable. Mechanically related variables should be interpreted as groups (geometry, concrete, and reinforcement), and a grouped-feature ablation or conditional SHAP analysis is required before making stable variable-level claims.

Agreement between grouped SHAP patterns and established mechanics is treated only as a plausibility check. It does not independently verify causal structural mechanisms, particularly because several interaction variables were engineered from those same mechanics.

### 3.14. Conformal Prediction for Uncertainty Quantification [29,30]

Absolute-residual split conformal prediction was applied to provide marginal prediction intervals. For the specimen-level benchmark, a calibration subset separate from model fitting and the 214-specimen withheld test set supplied the finite-sample residual quantile q, giving the interval Vpred ± q. The later source-disjoint global and adaptive intervals are reported as source-transfer diagnostics. The archived manuscript materials establish their final coverage and width values, but do not specify the exact source allocation used for conformal calibration or the complete adaptive scaling rule; these source-disjoint intervals are therefore treated as exploratory diagnostics rather than as a fully reproducible new conformal procedure.

The conformal prediction interval is defined as

$$\begin{array}{c} \mathrm{PI}=\left[V_{\mathrm{pred}}-q,\;V_{\mathrm{pred}}+q\right] \end{array}$$

where *PI* denotes the prediction interval, *V*_pred_ is the predicted punching shear capacity, and *q* is the conformal quantile estimated from the calibration residuals.

The conformal quantile is expressed as

$$q=Q_{1-\alpha }\left({\left\{|V_{\exp,i}-V_{\mathrm{pred},i}|\right\}}_{i=1}^{N_{\mathrm{cal}}}\right)$$

where *Q*_1−*α*_(⋅) denotes the empirical (1 − *α*)-quantile of the absolute calibration residuals, *V*_exp,*i*_ is the experimentally measured punching shear capacity of the *i*^th^ calibration specimen, *V*_pred,*i*_ is the corresponding model prediction, *N*_cal_ is the total number of calibration samples, and *α* is the significance level corresponding to the desired confidence level.

For ncal calibration residuals and miscoverage level α, the finite-sample quantile level was ceil[(ncal + 1)(1 − α)]/ncal. The 95% interval used the corresponding empirical quantile of absolute calibration residuals, with random_state = 42. $n_{\mathrm{cal}\;}\alpha q_{\mathrm{level}}=\min\left(1,\frac{\left\lceil \left(n_{\mathrm{cal}}+1\right)\left(1-\alpha \right)\right\rceil }{n_{\mathrm{cal}}}\right)$.

The withheld test set was not used for calibration.

Coverage and mean prediction-interval width were used to assess marginal calibration and sharpness. The original specimen-level split and the source-disjoint diagnostic are reported separately; neither aggregate coverage result guarantees connection-specific, loading-specific, or capacity-conditional coverage.

The implemented absolute-residual method produces a symmetric global interval of constant half-width q around each point prediction. It provides marginal coverage under exchangeability, not specimen-specific width or guaranteed conditional coverage.

The conformal procedure therefore supplements point predictions with marginally calibrated intervals under exchangeability; it does not guarantee conditional or specimen-specific coverage.

### 3.15. Monte Carlo Robustness Analysis

A 5000-realization stress test independently varied selected inputs within ±15% of nominal values. These bounded perturbations are not test-calibrated probability distributions and omit cross-variable dependence; outputs are therefore robustness scenarios rather than failure probabilities.

The perturbations were sampled independently using bounded marginal ranges. The ±15% range was not derived from test-specific distributions, and cross-variable dependencies were not modeled. Accordingly, the results are interpreted as scenario-based sensitivity to simultaneous perturbations, not as failure probabilities or a formal reliability analysis.

For each realization, a perturbed input vector was generated and evaluated using the optimized XGBoost model to obtain the corresponding predicted punching shear capacity. The statistical characteristics of the simulated predictions were subsequently quantified using the mean value, standard deviation, coefficient of variation (COV), and the 5th and 95th percentiles.

The coefficient of variation was calculated as

$$\begin{array}{c} \mathrm{COV}(\%)=\frac{\sigma }{\mu }\times 100 \end{array}$$

where *μ* denotes the mean predicted punching shear capacity and *σ* represents the corresponding standard deviation.

Probability density functions and cumulative distribution functions were analyzed to characterize the predicted-capacity distributions and evaluate the sensitivity of the optimized model to the prescribed bounded input perturbations. The ±15% ranges were not calibrated from empirical probability distributions, and cross-variable dependence was not modeled. The results therefore represent scenario-based sensitivity rather than physical uncertainty distributions, failure probabilities, or formal structural reliability.

The bounded perturbation analysis complements conformal prediction by examining how simultaneous input changes propagate through the fitted model. Together, they characterize residual uncertainty and scenario sensitivity without being presented as a formal reliability analysis.

### 3.16. Engineering Safety Assessment

Post-punching response was evaluated separately using peak load, residual-load ratio, crack bridging, and the Collapse-Arrest Index (CAI), defined as post-peak absorbed energy divided by total absorbed energy. Higher CAI denotes a larger post-peak energy fraction, not necessarily greater peak resistance.

To provide a quantitative measure of post-failure structural resilience, a Collapse-Arrest Index (CAI) is introduced and defined as

$$\mathrm{CAI}=\frac{E_{\mathrm{post}\backslash \mathrm{mbox}-\mathrm{peak}}}{E_{\mathrm{total}}}$$

where *E*_post\mbox−peak_ is the energy absorbed after reaching the peak load, and *E*_total_ is the total energy absorbed throughout the loading test.

The proposed index provides a normalized measure of the proportion of energy dissipated during the post-peak stage and therefore reflects the ability of the structural system to sustain load after punching failure. Higher CAI values indicate greater post-failure resilience and an improved capacity to arrest localized punching damage.

In addition to CAI, the experiment considered residual load ratio, post-peak deformation, crack bridging, and failure localization, providing a broader description of post-punching integrity than peak capacity alone.

Accordingly, the experimental program complements the ultimate-resistance prediction study by addressing a separate post-failure performance objective. It provides mechanics-consistency and case-specific post-peak observations for the tested configurations but does not verify machine-learning predictions, establish causal SHAP attribution, or demonstrate general design applicability.

## 4. Results and Discussion

### 4.1. Performance of Machine Learning Models

**Figure 4:**
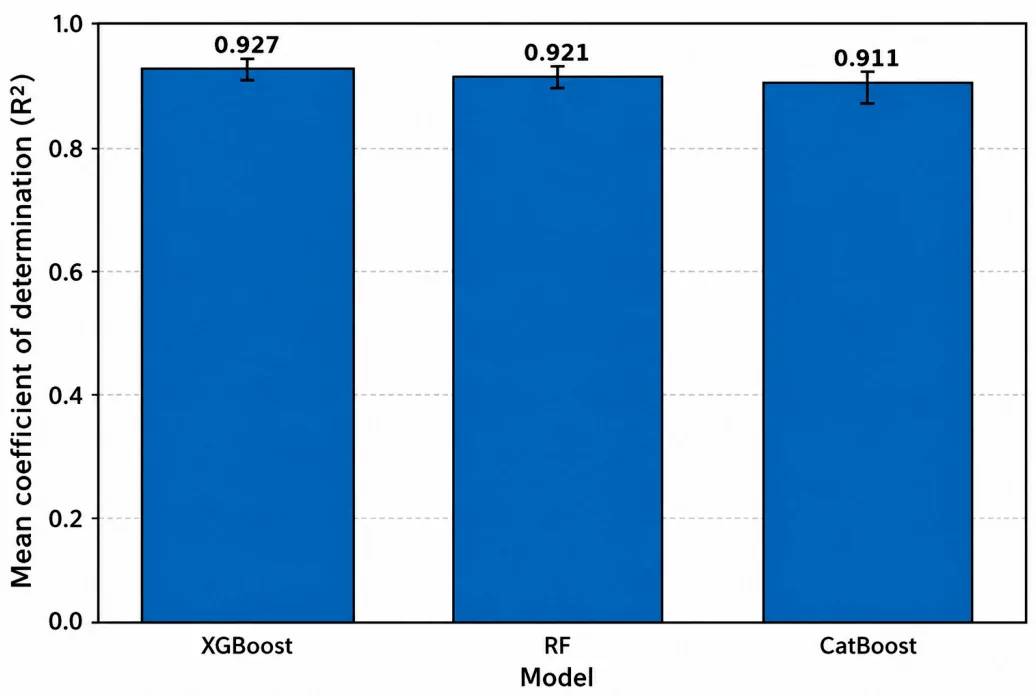
Five-fold cross-validation performance of the investigated ML models (mean R^2^ ± standard deviation).

The comparative five-fold specimen-level cross-validation results for XGBoost, Random Forest, and CatBoost are summarized in Table 8 and Figure 4.

The between-fold standard deviations of R^2^ were 0.015, 0.014, and 0.034 for XGBoost, Random Forest, and CatBoost, respectively (Table 8; Figure 4), indicating relatively limited variation across the comparative specimen-level partitions. The XGBoost value R^2^ = 0.927 in Table 8/Figure 4 is the mean from the comparative three-model cross-validation workflow, whereas R^2^ = 0.932 is the separately reproduced specimen-random five-fold benchmark used in the later source-separation analysis. They are therefore reported as results from distinct evaluation runs and should not be interpreted as the same numerical estimate. Because neither specimen-level workflow grouped records by source publication or campaign, both characterize within-database specimen-level performance rather than transfer to unseen experimental programs.

### 4.2. Withheld Specimen-Level Test Performance

**Figure 5:**
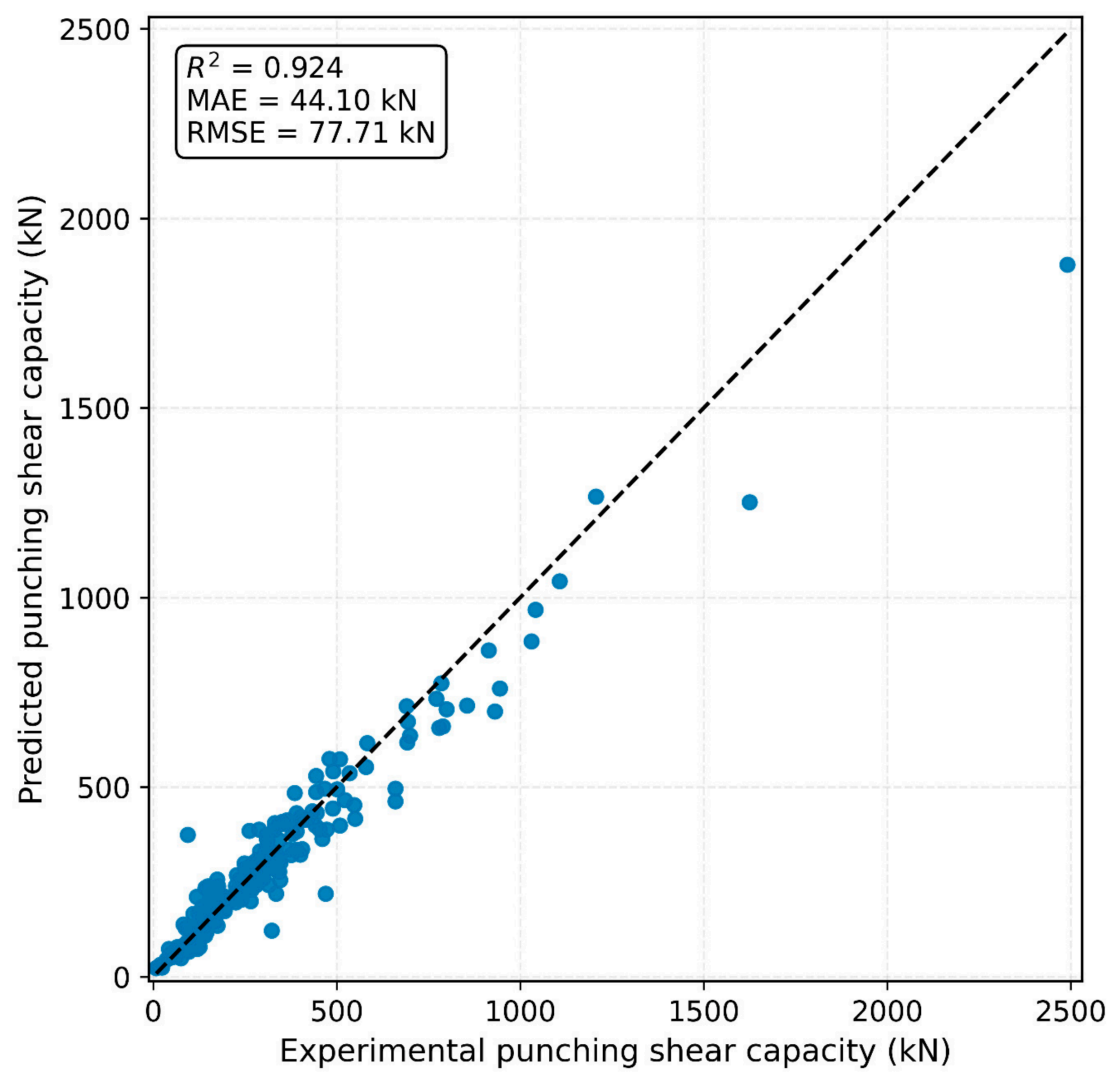
Comparison between experimentally measured and predicted punching shear capacities for the withheld specimen-level test dataset. The dashed line represents the line of perfect agreement (y = x).

On the 214 withheld specimens, XGBoost achieved R^2^ = 0.924, MAE = 44.10 kN, RMSE = 77.71 kN, MBE = 12.01 kN, MPE = −3.90%, and MAPE = 17.39% (Figure 5). These aggregate results indicate limited mean bias, although subgroup and source-level bias require separate assessment.

### 4.3. Residual Diagnostics and Bias Assessment

**Figure 6:**
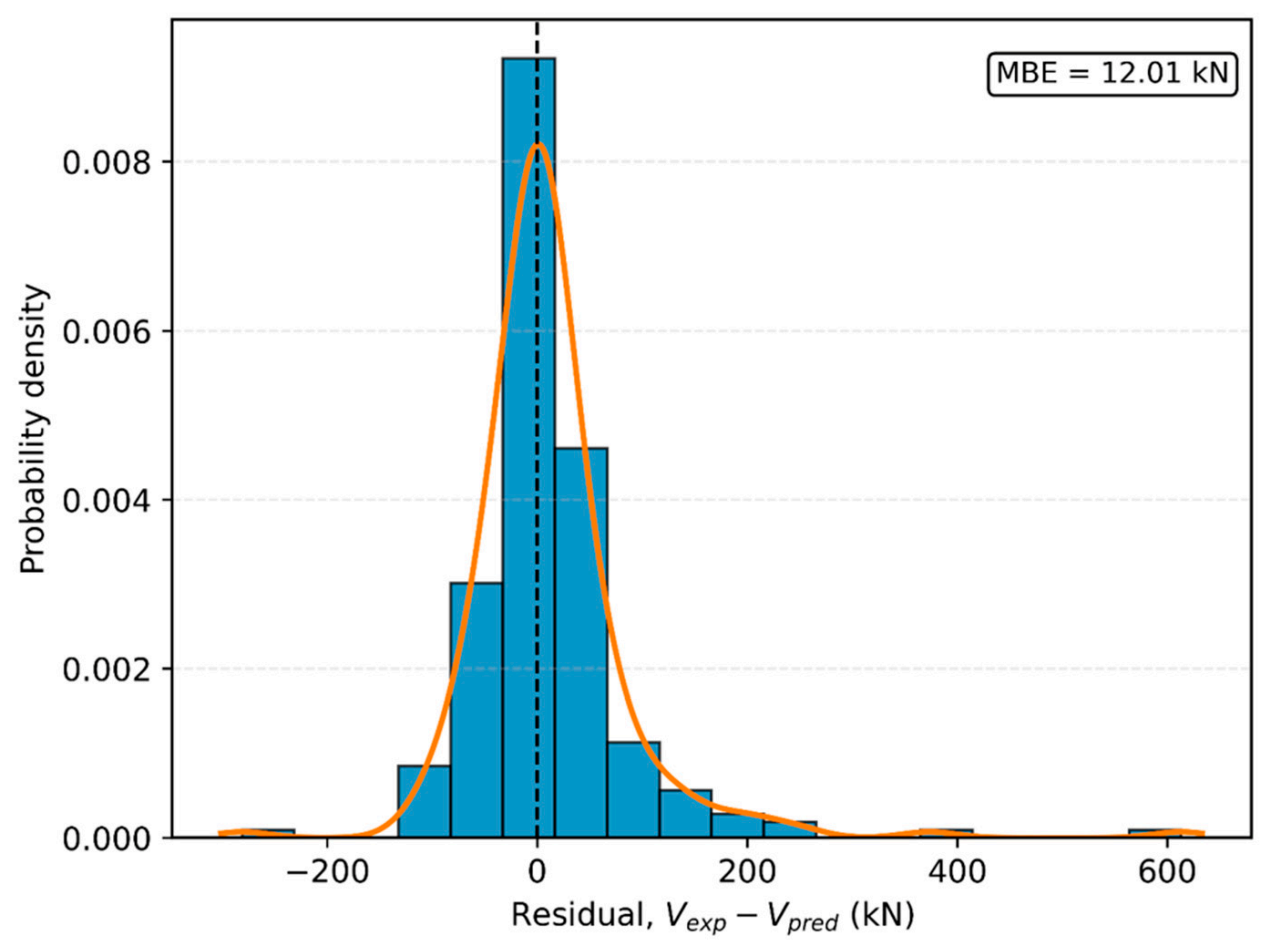
Distribution of prediction residuals for the withheld specimen-level test dataset. The orange solid line represents the kernel density estimate (KDE), while the black dashed line indicates zero residual.

**Figure 7:**
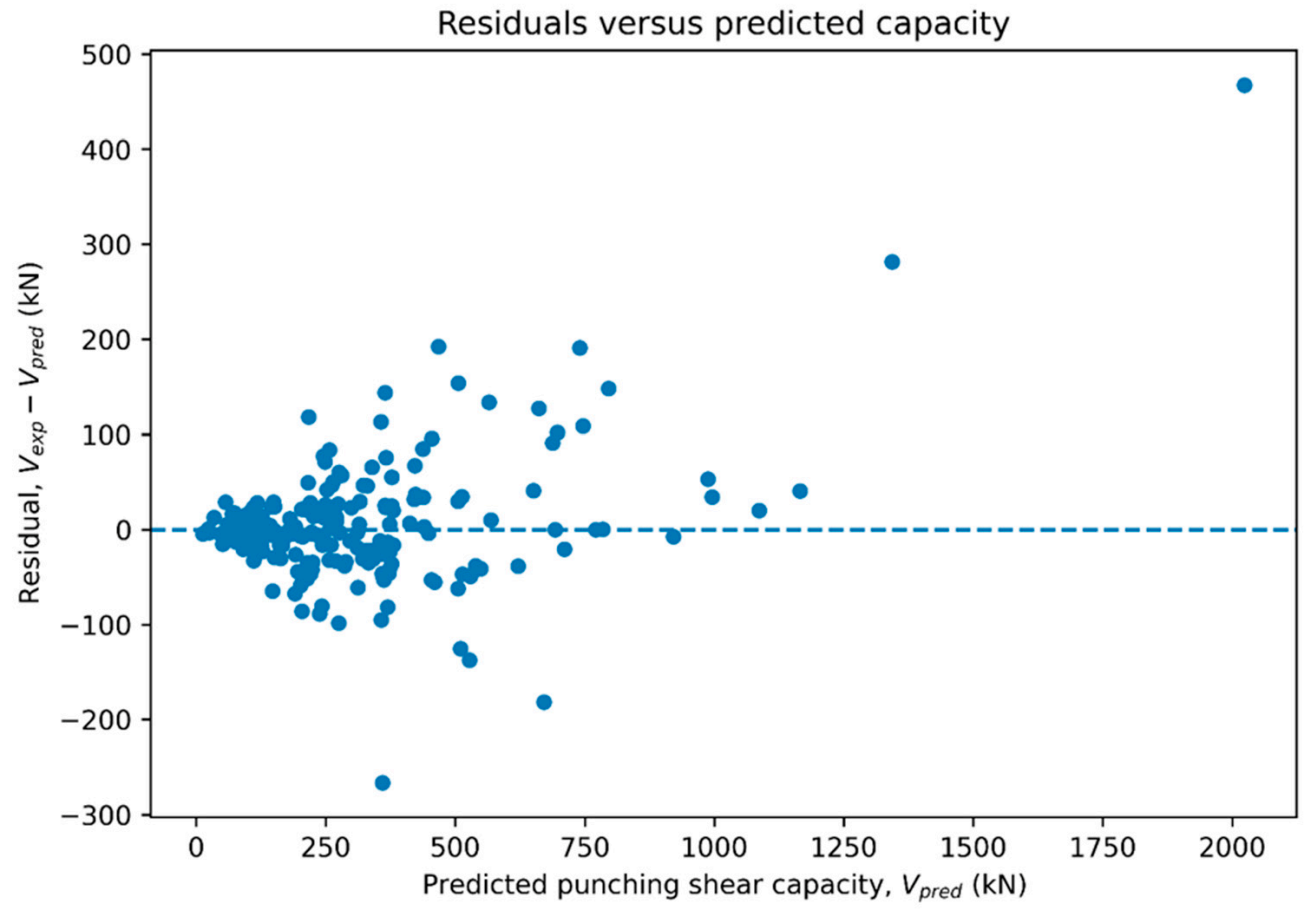
Residuals versus predicted punching shear capacity for the optimized XGBoost model. The blue dashed line indicates zero residual (perfect agreement between predicted and experimental capacities).

Residual diagnostics for the 214 withheld specimen-level test records are summarized in Table 9 and Figure 6 and Figure 7. The model gave MBE = 12.01 kN, MPE = −3.90%, and MAPE = 17.39%; residuals were concentrated around zero, with greater dispersion at higher capacities.

Figure 6 shows the residual distribution, while Figure 7 shows residuals versus predicted capacity. These diagnostics describe the withheld specimen-level benchmark only and do not establish source-level or subgroup-uniform error behavior.

Taken together, the residual diagnostics indicate limited aggregate bias but increasing dispersion for a small number of high-capacity cases; subgroup and source-separated performance are therefore evaluated separately in the following section.

### 4.4. Source-Grouped Generalization Performance

**Figure 8:**
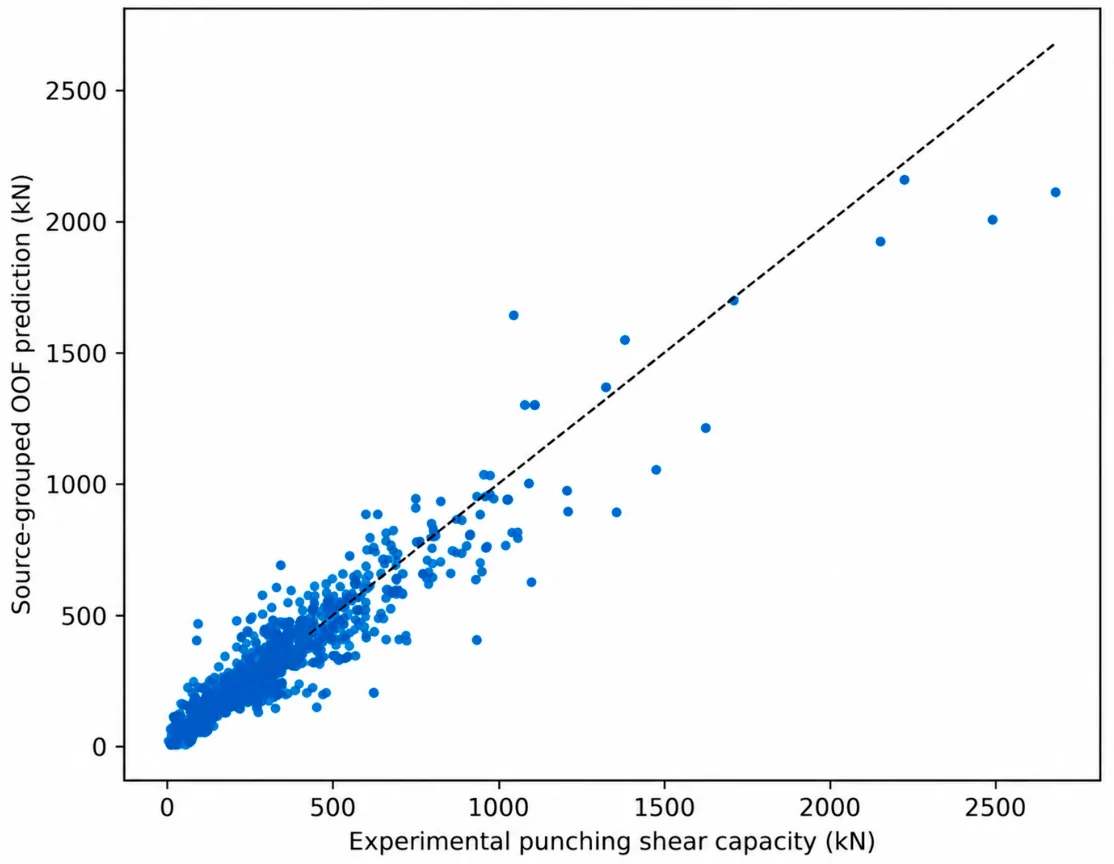
Source-grouped out-of-fold observed versus predicted capacity.

**Figure 9:**
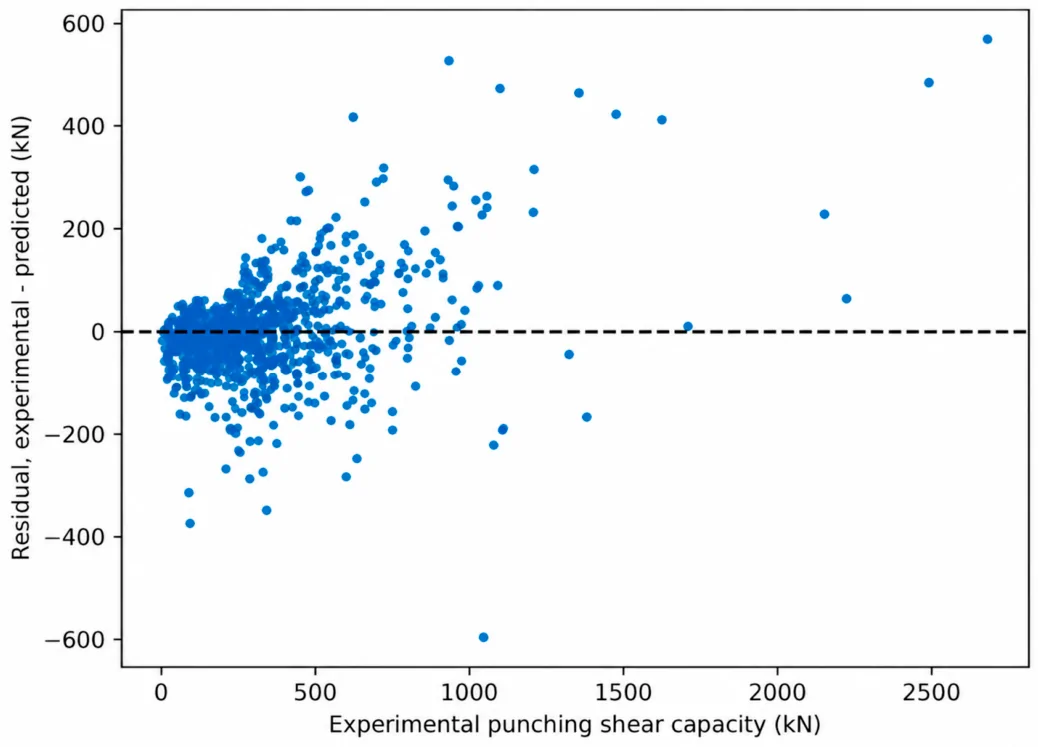
Source-grouped out-of-fold residuals versus observed capacity.

Source-grouped five-fold out-of-fold prediction using the globally selected grouped-grid configuration produced R^2^ = 0.881, MAE = 60.71 kN, RMSE = 93.22 kN, MBE = −1.21 kN, MPE = −13.97%, and MAPE = 29.84%. The reproduced specimen-random five-fold benchmark gave R^2^ = 0.932, MAE = 42.68 kN, and RMSE = 70.54 kN. The leakage-safe nested source-grouped audit, reported separately in Section 4.11, gave the more conservative pooled estimate R^2^ = 0.858, MAE = 66.38 kN, and RMSE = 101.80 kN. The separate source-disjoint test, with model selection performed using the 88 training sources and evaluation on 23 unseen source labels, gave R^2^ = 0.793, MAE = 55.38 kN, RMSE = 83.73 kN, and MAPE = 23.05%. The lower source-disjoint R^2^ does not correspond to a monotonic worsening of MAE or RMSE relative to GroupKFold because R^2^ and absolute-error metrics depend on the response distribution and composition of each evaluation set. GroupKFold already enforces source separation within each fold; the additional role of the source-disjoint holdout is that its 23 literature sources remain outside model selection and are used only for final held-out-study evaluation. These results quantify protocol-dependent transfer under the stated partitions and do not establish universal external validity. Table 10 compares the protocols, while Figure 8 and Figure 9 show the grouped out-of-fold predictions and residuals, respectively.

Connection-specific grouped results expose the database imbalance. Interior specimens (n = 983) achieved R^2^ = 0.886, MAE = 59.89 kN, RMSE = 92.90 kN, and MAPE = 26.83%; edge specimens (n = 51) achieved R^2^ = 0.309, MAE = 82.39 kN, RMSE = 111.11 kN, and MAPE = 50.85%; and corner specimens (n = 34) achieved R^2^ = −0.851, MAE = 51.94 kN, RMSE = 70.35 kN, and MAPE = 85.18%. Negative corner R^2^ indicates that the grouped out-of-fold predictions were worse than predicting the corner-subgroup mean. The substantially higher relative errors for edge and especially corner connections further demonstrate that aggregate model performance should not be interpreted as uniform predictive capability across connection types. Sparse edge/corner data, eccentric moment transfer, truncated control perimeters, and campaign-specific boundary conditions therefore prevent credible broad predictive claims for these configurations.

Bootstrap confidence intervals were additionally calculated for the connection-specific grouped out-of-fold metrics. For interior connections, R^2^ = 0.886 (95% bootstrap CI = 0.857 to 0.907) and MAE = 59.89 kN (95% bootstrap CI = 55.64 to 64.39 kN). For edge connections, R^2^ = 0.309 (95% bootstrap CI = −0.207 to 0.611) and MAE = 82.39 kN (95% bootstrap CI = 62.89 to 103.33 kN). For corner connections, R^2^ = −0.851 (95% bootstrap CI = −3.123 to 0.113) and MAE = 51.94 kN (95% bootstrap CI = 36.39 to 68.06 kN). The substantially wider intervals for the sparse edge and corner subsets reinforce that these subgroup estimates are less stable than those obtained for interior connections.

The fixed-hyperparameter grouped ablation showed a modest contribution from engineered terms: R^2^ changed from 0.880 to 0.881 and MAE from 62.51 to 60.71 kN. Grouped held-out permutation analysis ranked geometry/loading first, followed by longitudinal reinforcement, fibers, and concrete strength. These associations are mechanically plausible but not causal because correlated original and engineered variables can exchange attribution. The standardized fold-specific five-nearest-neighbor screen gave MAE = 54.79 kN within and 87.88 kN outside the training-density envelope. Although outside-domain R^2^ was numerically higher, R^2^ is composition-dependent and does not contradict the materially larger absolute errors; the domain flag is therefore based on data support and error magnitude, not R^2^ alone.

### 4.5. Benchmark Comparison with Current Design Provisions

XGBoost was benchmarked against the nominal resistance equations of ACI 318-19 and Eurocode 2 using the same 214-specimen withheld set. Strength-reduction and material partial factors were omitted so that the comparison addresses nominal prediction error rather than design safety. fib Model Code 2010 and CSCT were not implemented numerically because the deformation and slab-rotation information required for consistent application was unavailable for much of the database.

**Figure 10:**
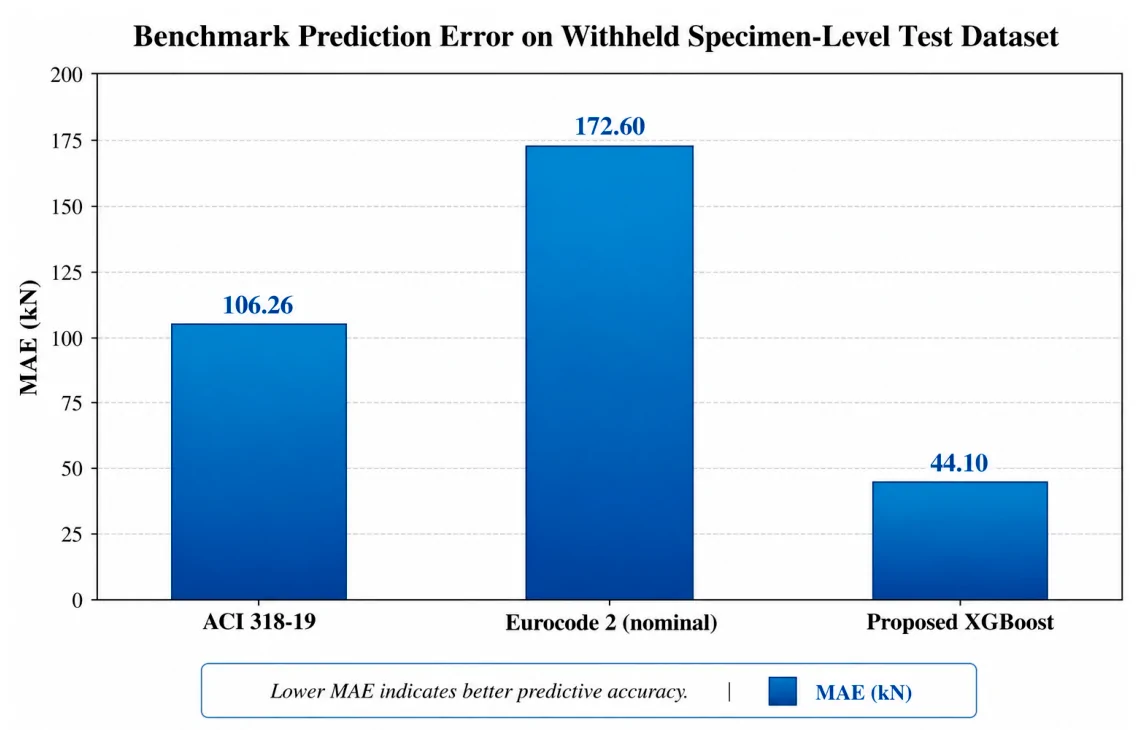
Benchmarking of predictive performance using mean absolute error (MAE) for ACI 318–19, Eurocode 2 (nominal), and the optimized XGBoost model on the withheld specimen-level test dataset (N = 214). Lower MAE values indicate better predictive accuracy.

Table 11 shows that XGBoost yielded the lowest prediction errors on this specimen-level benchmark (R^2^ = 0.924, MAE = 44.10 kN, RMSE = 77.71 kN, MAPE = 17.39%), compared with ACI 318-19 (0.762, 106.26 kN, 137.12 kN, and 47.70%) and Eurocode 2 (0.322, 172.60 kN, 231.71 kN, and 55.67%), respectively. Figure 10 presents the corresponding MAE benchmark (106.26, 172.60, and 44.10 kN, respectively), where lower MAE indicates better predictive agreement. These results quantify nominal predictive agreement only; they neither compare complete design systems nor imply replacement of code-based resistance, safety, detailing, or applicability requirements.

### 4.6. SHAP-Based Explainability Analysis

#### 4.6.1. Global and Grouped Feature Importance

**Figure 11:**
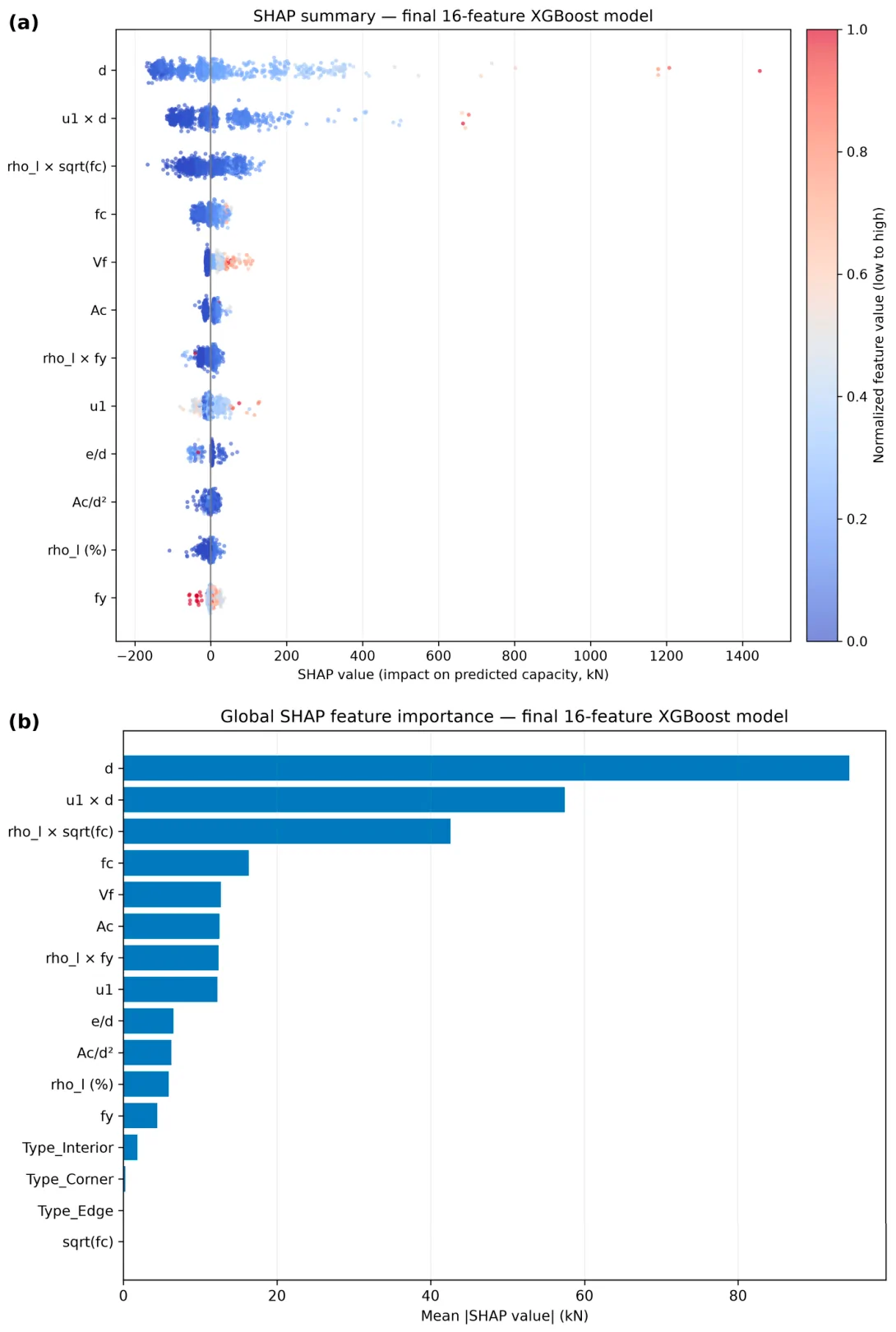
Global SHAP analysis of the selected 16-feature XGBoost configuration: (**a**) SHAP summary distribution showing the magnitude and direction of model-attributed contributions and (**b**) global feature-importance ranking based on mean absolute SHAP values.

Figure 11 presents the global SHAP distributions and the corresponding mean absolute SHAP importance values for the selected 16-feature XGBoost configuration. Because several engineered descriptors are deterministic combinations of the original variables, the importance assigned to individual features should not be interpreted independently. The results are therefore considered both at the individual-feature level and in terms of broader geometry, reinforcement, fiber, and concrete-related mechanisms.

The SHAP analysis indicates that geometry-related information dominates the fitted response, with effective depth and perimeter-related descriptors among the highest-ranked predictors, followed by reinforcement-concrete, concrete-strength, fiber, and loaded-area information. The direction of the principal associations is qualitatively consistent with established punching-shear mechanics. However, because several engineered descriptors are deterministic combinations of measured variables, attribution can be redistributed among correlated original and engineered predictors; individual SHAP rankings therefore cannot be interpreted as uniquely separable physical effects.

Connection-type indicators exhibited comparatively small global SHAP contributions, but this should not be interpreted as evidence that connection configuration is mechanically unimportant. The database is dominated by interior connections, while the source-grouped results show substantially weaker transfer for edge and corner configurations. Consistently, source-grouped permutation importance at the engineering-block level ranked geometry/loading first, followed by longitudinal reinforcement, fibers, and concrete-related information. The interpretability analysis is therefore used as a mechanics-consistency audit of predictive associations, not as causal identification of governing structural mechanisms.

#### 4.6.2. Local Interpretation

Dependence patterns revealed nonlinear associations and interactions among geometry, reinforcement, and concrete-related predictors. Larger d and u1d generally shifted predictions upward within the represented data range. These patterns support mechanics-consistency assessment but do not establish causal effects.

#### 4.6.3. Relationship with Current Design Practice

The grouped importance patterns are qualitatively consistent with the roles assigned to effective depth, control perimeter, concrete strength, and reinforcement in established punching-shear formulations. Because related mechanics-informed descriptors were deliberately included in the predictor set, this agreement is interpreted only as a plausibility check rather than independent physical validation.

### 4.7. Uncertainty Quantification Using Split Conformal Prediction

**Figure 12:**
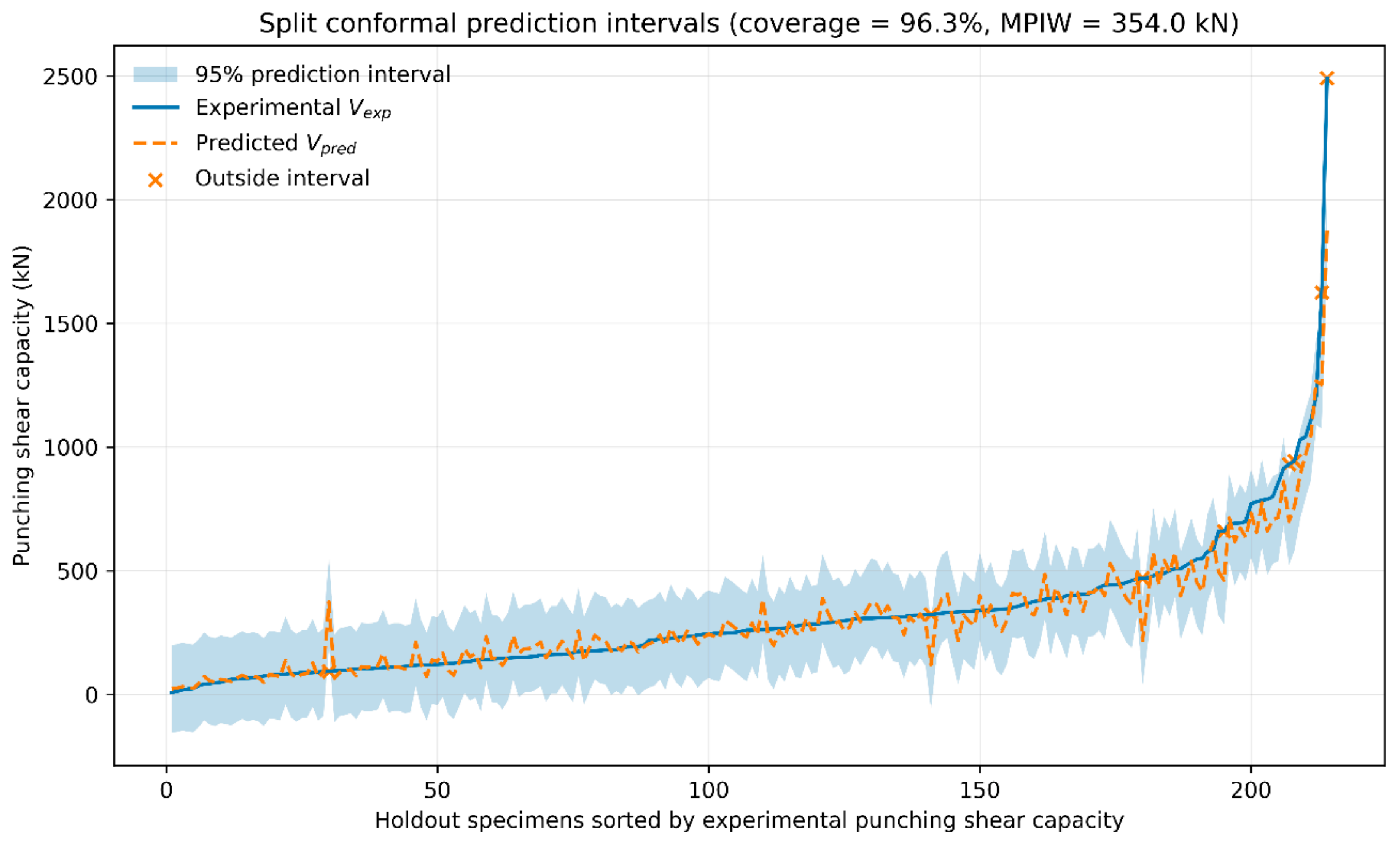
Split conformal prediction intervals obtained for the withheld specimen-level test dataset. The shaded region represents the 95% prediction interval, whereas the solid and dashed curves denote the experimentally measured and predicted punching shear capacities, respectively. The observed empirical coverage was 96.3%, with a mean prediction interval width (MPIW) of 354.0 kN.

Figure 12 reports the specimen-level split-conformal benchmark, which achieved 96.3% marginal coverage at a nominal 95% level with a constant total interval width of 354.04 kN (q = 177.02 kN). Under source-disjoint evaluation, the reported global diagnostic achieved 97.90% coverage with a mean width of 507.14 kN, while the exploratory adaptive diagnostic achieved 96.22% coverage with a larger mean width of 544.74 kN and therefore did not improve sharpness. Because the archived analysis does not fully specify the source-level calibration allocation and adaptive scaling rule, these source-disjoint values are interpreted as diagnostic evidence rather than as a separately reproducible conformal method. The source-disjoint test contained 237 interior, one edge, and no corner specimen, so configuration-conditional coverage cannot be estimated credibly for the sparse connection types.

### 4.8. Perturbation-Based Robustness Analysis

#### 4.8.1. Statistical Evaluation

**Figure 13:**
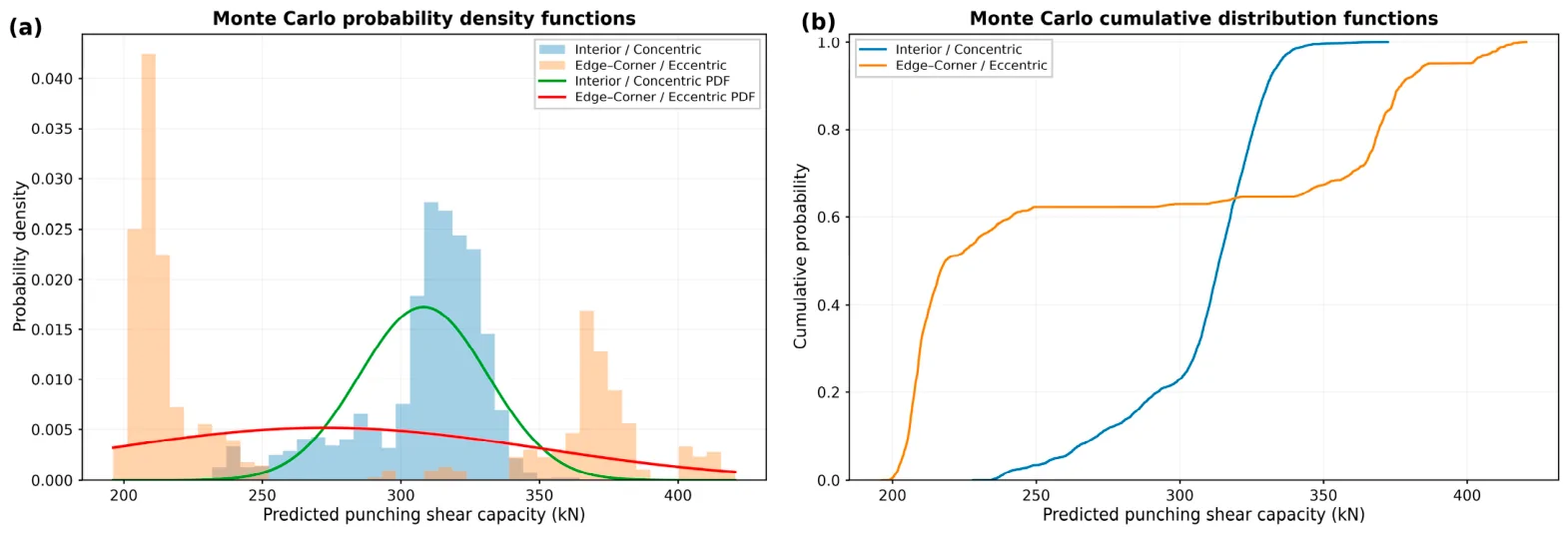
Monte Carlo robustness analysis: (**a**) probability density functions (PDFs) and (**b**) cumulative distribution functions (CDFs) of the predicted punching-shear capacity for the interior/concentric and edge–corner/eccentric scenarios.

Under the prescribed 5000 bounded perturbations, the interior/concentric scenario showed a mean predicted capacity of 308.20 kN and COV of 7.51%, whereas the edge/corner eccentric scenario showed 272.57 kN and 28.39%, respectively (Table 12; Figure 13). The wider response of the latter scenario indicates greater model sensitivity to the imposed input perturbations. These distributions represent bounded sensitivity diagnostics only and should not be interpreted as calibrated input uncertainty, failure probability, or structural reliability.

#### 4.8.2. Distribution of Perturbation Responses

Figure 13 presents the predicted punching-shear capacity distributions under bounded perturbations. The interior/concentric scenario shows a narrower distribution (COV = 7.51%), whereas the edge–corner/eccentric scenario exhibits greater dispersion (COV = 28.39%), indicating higher sensitivity to input perturbations. These distributions reflect model sensitivity rather than calibrated uncertainty or structural reliability.

### 4.9. Mechanics-Consistency Experimental Assessment

#### 4.9.1. Experimental Scope and Reporting

The separate nine-specimen program was used exclusively for complementary mechanics-consistency and post-failure assessment and not for predictive validation. Quantitative post-peak comparisons were restricted to the three specimens with complete digitized load–displacement records.

#### 4.9.2. Failure Mechanism and Structural Integrity

**Figure 14:**
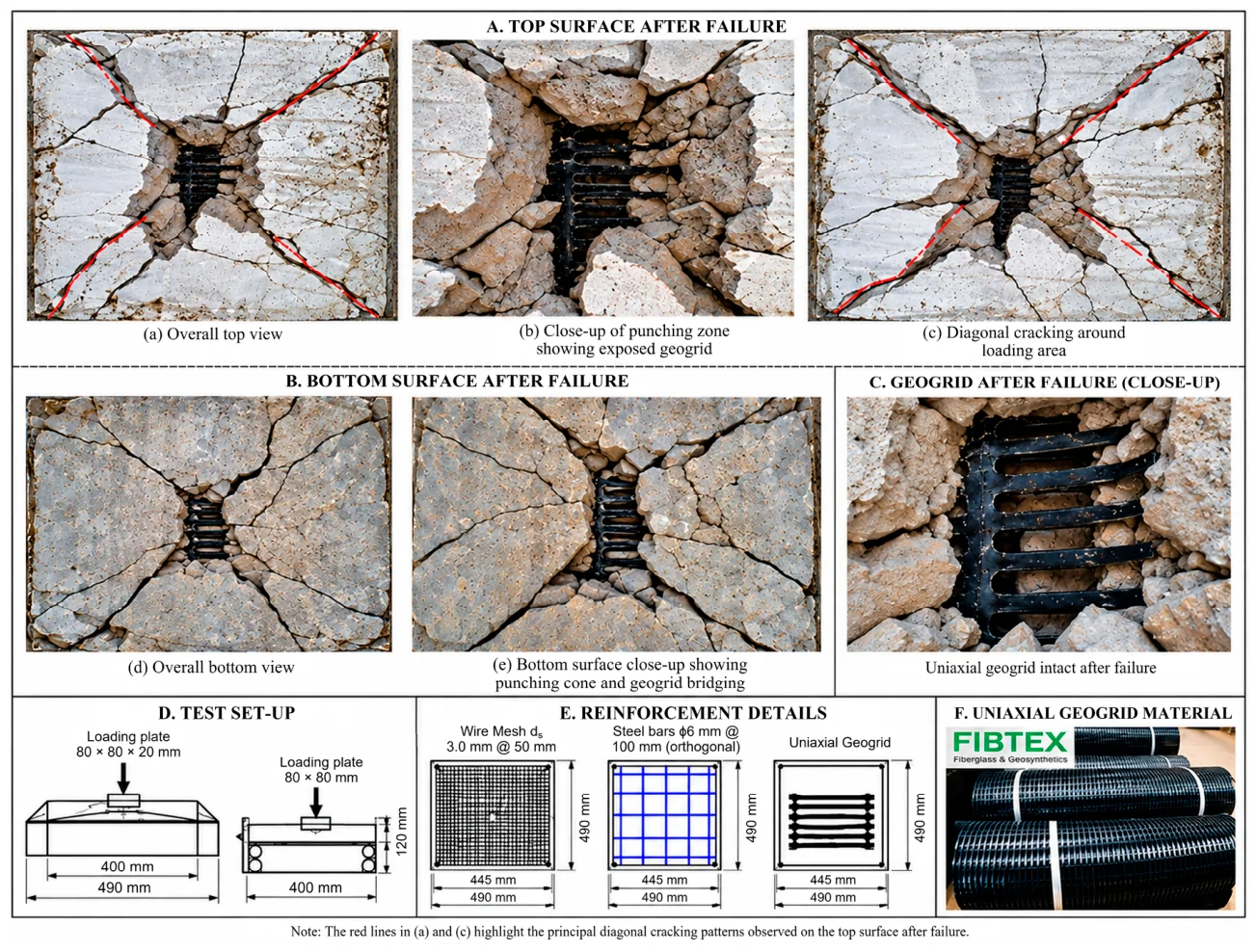
Representative failure patterns illustrating localized punching failure, crack propagation, punching cone formation, and crack-bridging action of the proposed uniaxial geogrid after testing.

Figure 14 illustrates representative localized punching damage, crack development, punching-cone formation, and post-failure engagement of the embedded uniaxial geogrid.

#### 4.9.3. Post-Punching Load–Displacement Response and CAI

**Figure 15:**
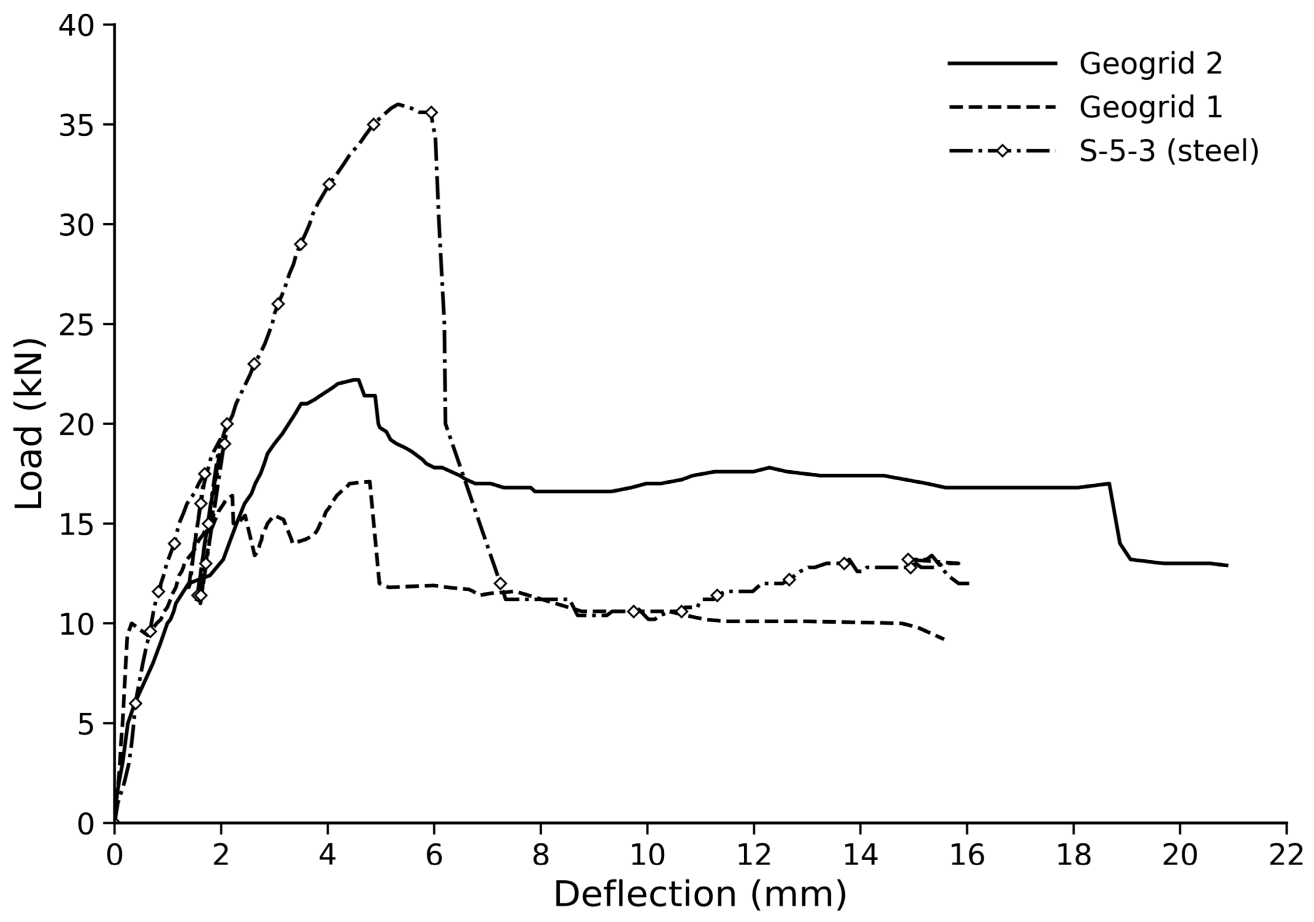
Load–displacement responses of the tested cementitious slabs illustrating the elastic response, peak punching load, and post-peak behavior of the proposed uniaxial geogrid reinforcement system.

Figure 15 presents the three specimens for which complete digitized post-peak load–displacement records were available. Quantitative CAI and residual-load comparisons are restricted to these three records; the remaining six tests are excluded from quantitative post-peak comparison because equivalent complete digitized records were unavailable.

Table 13 summarizes the post-peak metrics for the three specimens with complete digitized load–displacement records.

Among the three specimens with complete post-peak records, Geogrid-1 and Geogrid-2 reached peak loads of 17.1 and 22.2 kN, compared with 36.0 kN for Steel-S-5-3. Their residual-load ratios were nevertheless higher (0.538 and 0.581 versus 0.333), as were their CAI values (0.640 and 0.807 versus 0.560). Thus, the tested geogrid specimens exhibited greater normalized post-peak retention, not greater peak capacity. The observed crack-bridging behavior is consistent with continued tensile engagement after punching; however, three complete records are insufficient to establish population-level superiority, causality, or a design rule.

### 4.10. Engineering Implications

The results establish a hierarchy of predictive evidence rather than a monotonic error-degradation sequence. Specimen-level accuracy does not by itself establish cross-study transferability: R^2^ decreased under source-separated evaluation, but MAE and RMSE did not worsen monotonically between GroupKFold and the source-disjoint holdout because the evaluation sets differ in response distribution and composition. GroupKFold already isolates literature sources within folds, whereas the source-disjoint holdout additionally keeps the held-out studies outside model selection. The leakage-safe nested audit provides the most conservative source-grouped estimate and confirms that the central limitation is transfer across heterogeneous studies, particularly for sparsely represented edge and corner configurations. The source-aware learning curve further indicates that expanding independent source diversity is more consequential than increasing model complexity.

For engineering screening, model output should be interpreted jointly with applicability-domain status, predictive uncertainty, connection-specific evidence, mechanics-consistency diagnostics, and governing code requirements. SHAP attribution should be treated as associative, conformal intervals as marginal predictive uncertainty rather than reliability bounds, and code benchmarking as nominal-error context. The separate geogrid observations address post-punching integrity rather than predictive validation or ultimate-resistance superiority. Any prediction outside the represented domain or inconsistent with established mechanics or code checks requires source-level review rather than automatic acceptance.

### 4.11. Nested Bayesian Optimization and Model-Complexity Audit

Nested Optuna produced pooled source-grouped out-of-fold R^2^ = 0.858, MAE = 66.38 kN, RMSE = 101.80 kN, MAPE = 33.07%, MBE = 0.30 kN, and MPE = −14.05%. Across outer folds, mean R^2^ was 0.854 ± 0.026, MAE was 66.38 ± 9.74 kN, and RMSE was 100.43 ± 18.60 kN. Selected tree counts ranged from 450 to 1413 (median 984), depths from 2 to 6, and learning rates from 0.0172 to 0.1071. This variation indicates source-fold sensitivity rather than a universal optimum.

The leakage-safe nested Optuna audit yielded pooled R^2^ = 0.858, MAE = 66.38 kN, RMSE = 101.80 kN, and MAPE = 33.07%. The grouped GridSearch result (R^2^ = 0.881, MAE = 60.71 kN, RMSE = 93.22 kN, MAPE = 29.84%) used hyperparameters selected globally with grouped cross-validation and is therefore retained as a tuned grouped estimate rather than the primary unbiased estimate of source-grouped generalization. The nested result is emphasized when discussing validation rigor because each outer fold remained untouched during hyperparameter selection. Table 14 compares the protocols; the difference should not be interpreted as evidence that either optimization algorithm is intrinsically superior.

**Figure 16:**
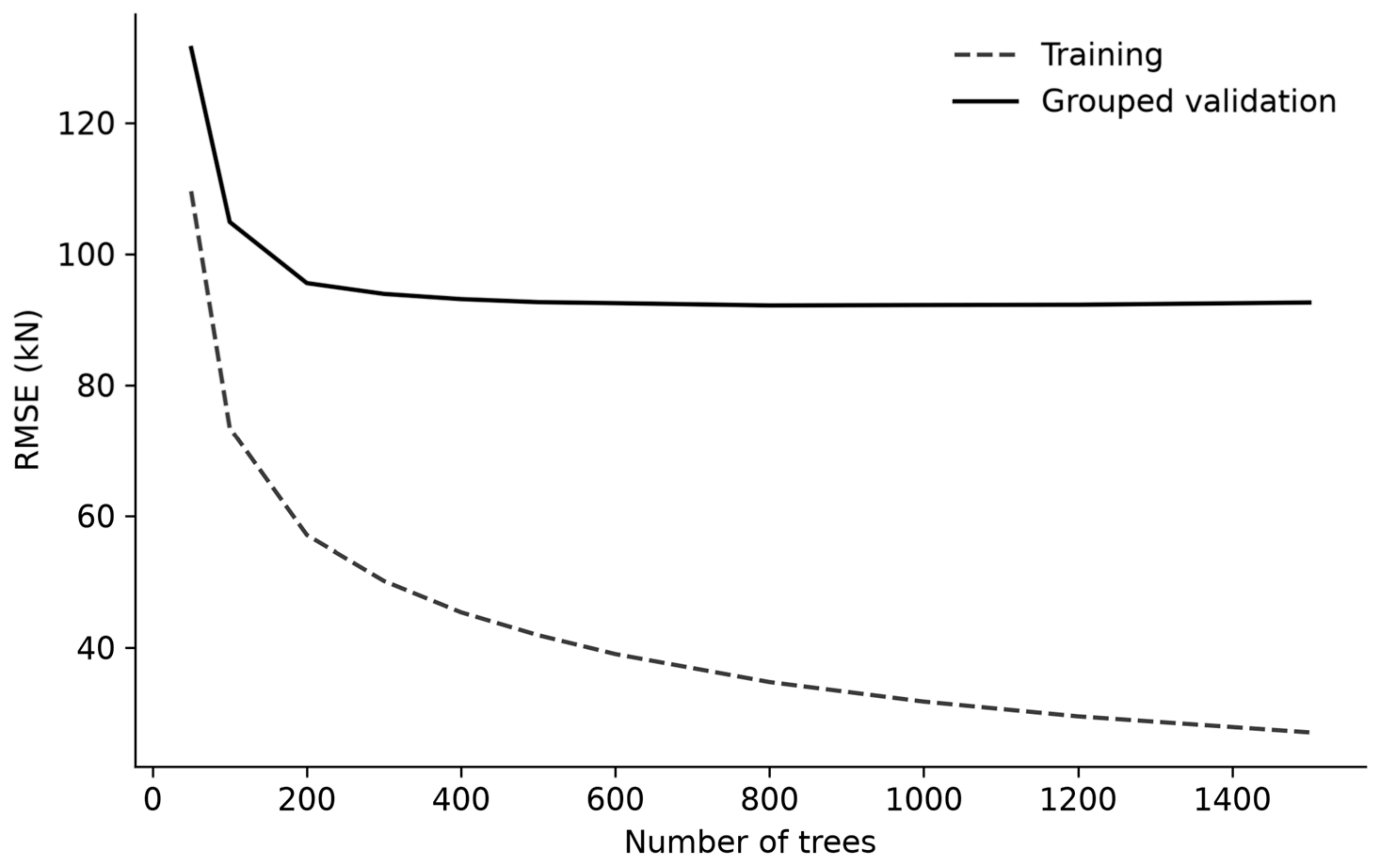
Training and source-grouped validation RMSE versus estimator count with the grouped-grid configuration otherwise fixed. The curve tests model-size sensitivity and does not prove absence of overfitting.

**Figure 17:**
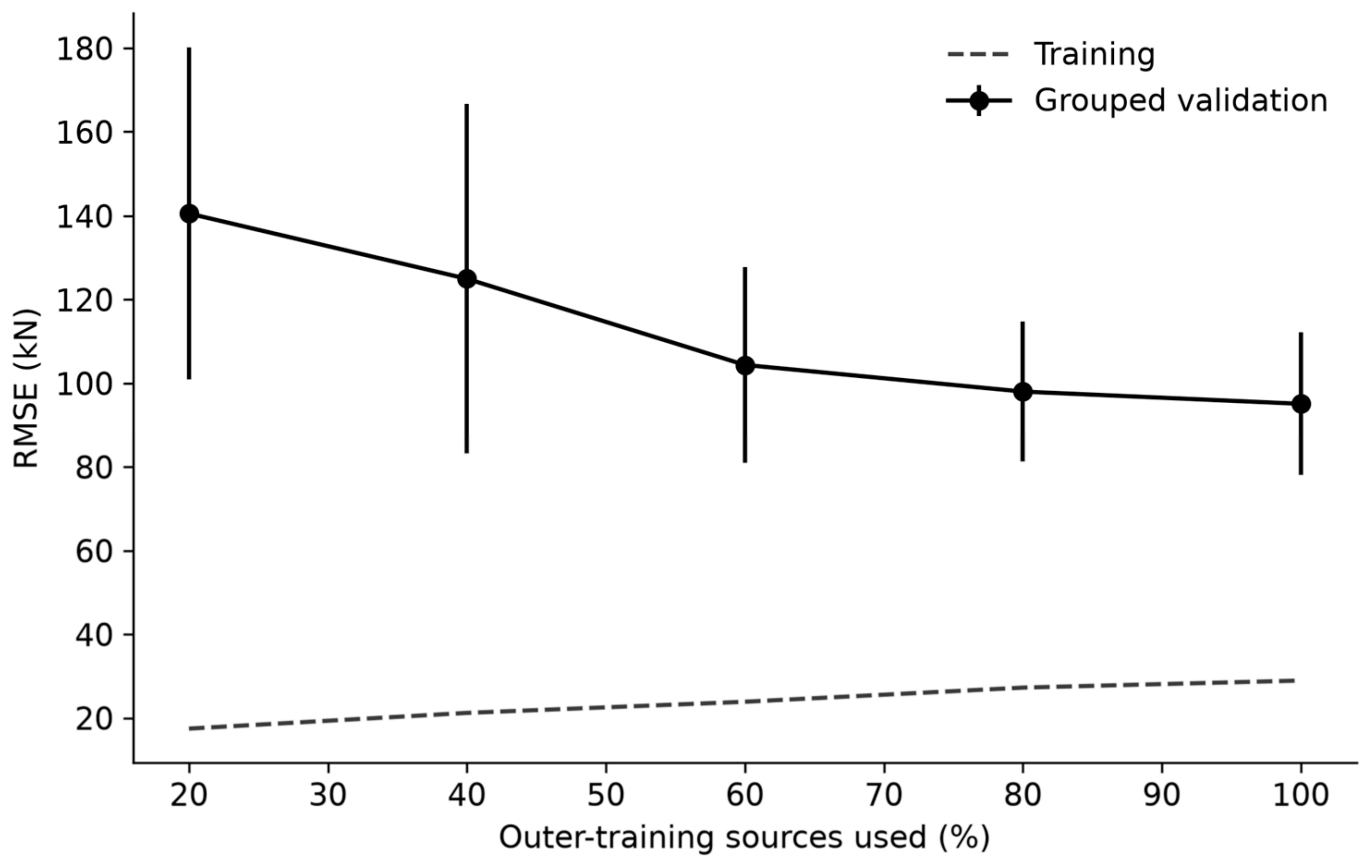
Source-aware learning curve based on repeated fractions of outer-training sources. Error bars show variation across source-separated evaluations.

Tree-count sensitivity was evaluated directly under source-grouped validation (Figure 16). Mean validation RMSE decreased from 131.39 kN at 50 trees to 92.15 kN at 800 trees, then remained nearly flat at 92.21, 92.25, and 92.59 kN for 1000, 1200, and 1500 trees. Training errors continued to decrease, but no material validation deterioration occurred at 800 trees. Thus, estimator count alone did not establish overfitting in this dataset. The source-aware learning curve (Figure 17) reduced grouped-validation RMSE from 140.58 kN at 20% of training sources to 95.10 kN at 100%, indicating that additional independent studies are more valuable than further model complexity.

Nested Optuna subgroup performance remained imbalanced: interior R^2^ = 0.863, MAE = 65.98 kN, RMSE = 102.04 kN, and MAPE = 30.35%; edge R^2^ = 0.222, MAE = 88.38 kN, RMSE = 117.95 kN, and MAPE = 46.63%; corner R^2^ = −0.349, MAE = 45.02 kN, RMSE = 60.05 kN, and MAPE = 91.52%. These results reinforce, rather than remove, the edge/corner applicability warning.

### 4.12. Engineering Screening and Implemented GUI Prototype

To demonstrate the engineering-screening pathway in executable form, a lightweight Streamlit graphical interface was developed and supplied as Supplementary Software (see Supplementary Materials). The implemented sequence is engineering inputs; unit and raw-range validation; deterministic feature construction; applicability-domain check; XGBoost point prediction; reference marginal conformal uncertainty display; connection-type warning; nominal ACI 318-19/Eurocode 2 benchmark context; and mandatory engineering reviews. The interface is intended for research screening and does not replace ACI 318, Eurocode 2, safety or partial factors, detailing requirements, load combinations, or professional judgment.

The operational XGBoost bundle uses the grouped-grid configuration and was refit on all 1068 QC records solely to make the software functional. This full-data refit is not additional validation evidence; all performance claims remain tied to the specimen-random, source-grouped, source-disjoint, and nested evaluations reported above. The interface calculates standardized mean five-nearest-neighbor distance and applies a full-database deployment-screening threshold of 1.903312, defined as the 95th percentile of self-excluded training distances. This threshold is a prototype screening diagnostic, not a new validation-performance statistic or a replacement for the fold-specific analysis.

The displayed ±177.02 kN uncertainty band is the manuscript reference split-conformal half-width associated with 96.3% observed marginal coverage at a nominal 95% level. It is not a deployment-recalibrated interval, subgroup-conditional guarantee, or structural-reliability result. The GUI presents the manuscript nominal code-benchmark metrics only; it does not calculate per-case ACI 318-19 or Eurocode 2 resistance because complete code-specific geometry, eccentricity treatment, safety formats, detailing checks, and scope verification are not implemented. The Supplementary Software package includes the Streamlit application, operational model bundle, QC data copy, requirements file, README, and methodology note; software checks are documented in the supplementary package rather than treated as research results in the manuscript.

**Figure 18:**
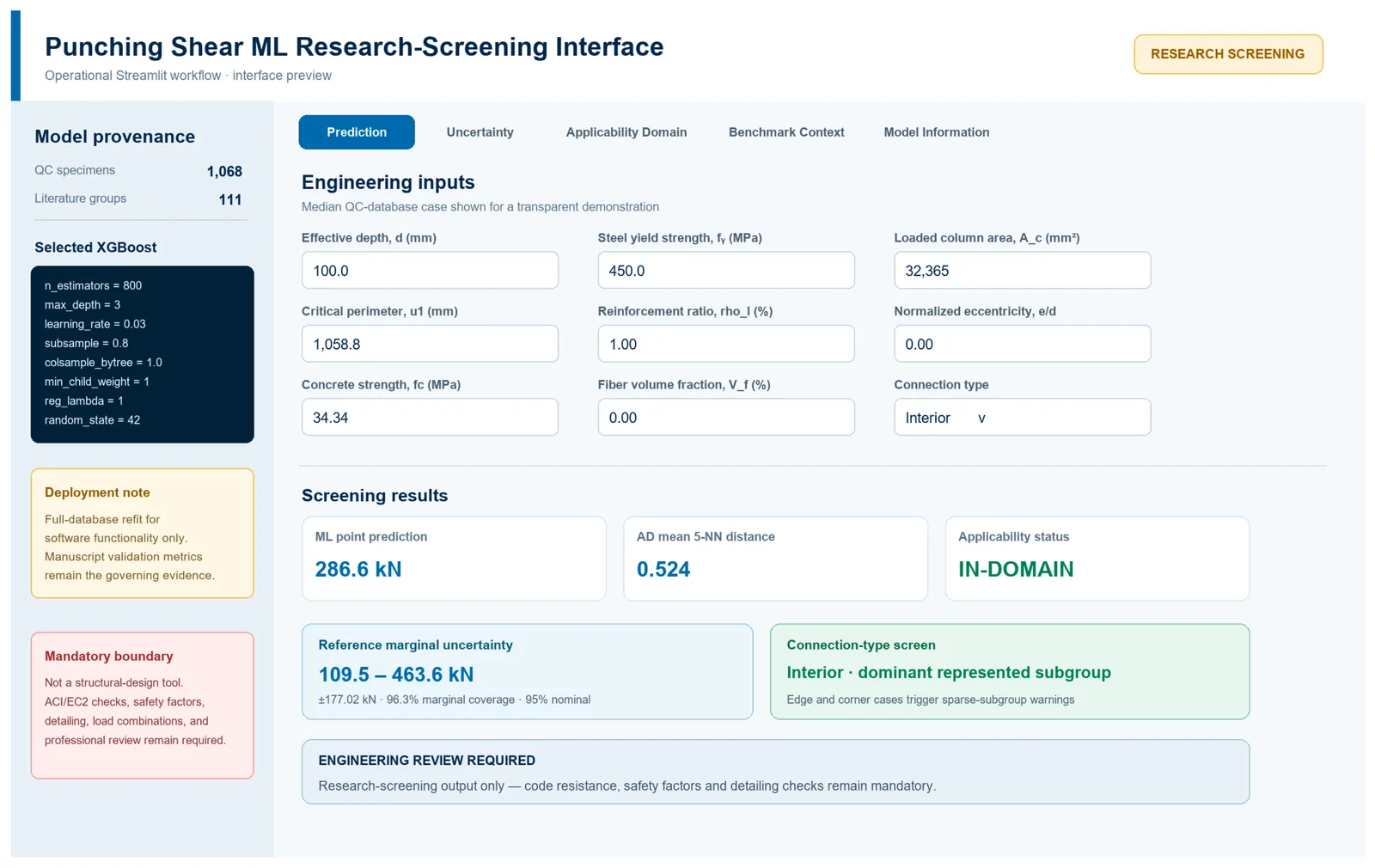
Implemented Streamlit research-screening interface for a representative median database case. The displayed output is a screening result, not a structural-design resistance; code checks, safety factors, detailing requirements, load combinations, and professional review remain mandatory.

Figure 18 presents the implemented research-screening interface for a representative median database case. The interface integrates the point prediction, applicability-domain status, reference marginal uncertainty, subgroup warning, and the mandatory engineering-review boundary.

### 4.13. Limitations

The analysis represents 111 literature sources only, and transfer beyond them is not established. Edge and corner samples remain scarce, with weak or negative grouped R^2^, and nested Optuna parameters vary across source folds. Exact selected legacy specimen-random RF and CatBoost hyperparameters were not archived, so their comparative results are not claimed to be independently reproducible from the supplied package; reproducibility claims are centered on the documented XGBoost workflows. SHAP and grouped importance remain associative under correlated predictors. Conformal intervals provide marginal, not conditional, coverage; the adaptive diagnostic did not improve sharpness, and the archived materials do not fully specify the source-level calibration allocation or adaptive scaling rule for the source-disjoint conformal diagnostics. Applicability-domain limits depend on training density, and absolute error increased materially outside that envelope. Independent ±15% perturbations are bounded sensitivity scenarios, not calibrated reliability analysis. Code comparisons concern nominal resistance only. The nine tests are separate mechanics experiments, with quantitative post-peak evidence limited to three complete records. The implemented GUI is a research-screening prototype and has not undergone prospective deployment, human-factors, or code-software verification. No design replacement is claimed.

## 5. Conclusions

This study developed an explainable machine-learning workflow for estimating punching shear capacity within the represented literature domain. Source-grouped validation, grouped interpretation, marginal uncertainty quantification, bounded sensitivity analysis, and a complementary geogrid experiment were used to define both capability and limitation; none constitute formal structural reliability verification.

The principal conclusions are summarized as follows.

1. A quality-controlled database of 1068 specimens from 111 verbatim source groups provides a broad but imbalanced literature-derived basis for model development and source-separated evaluation; 983 records are interior, 51 edge, and 34 corner connections.
2. XGBoost achieved R^2^ = 0.932 under the reproduced specimen-random five-fold benchmark. The tuned grouped-grid analysis gave R^2^ = 0.881, whereas the leakage-safe nested source-grouped audit gave R^2^ = 0.858; the latter is the more conservative estimate because hyperparameter selection was confined to inner source-grouped folds. The source-disjoint held-out-study evaluation gave R^2^ = 0.793. Because MAE and RMSE did not worsen monotonically between the grouped and source-disjoint sets, these results should be interpreted as protocol- and composition-dependent evidence of transfer rather than as a monotonic degradation sequence.
3. GroupKFold already prevents source overlap between training and validation within each fold. The source-disjoint holdout adds a different evidential constraint by keeping 23 complete literature sources outside model selection and using them only for held-out-study evaluation. This distinction, together with the nested audit, defines the validation-centered contribution of the study.
4. Grouped SHAP patterns emphasized geometry, concrete strength, and reinforcement-related variables. Their qualitative agreement with established mechanics supports plausibility but not causal or uniquely allocated feature attribution.
5. The specimen-level split-conformal benchmark achieved 96.3% marginal coverage with a constant total width of 354.04 kN. Under source-disjoint evaluation, the global interval achieved 97.90% coverage with a mean width of 507.14 kN, while the adaptive variant achieved 96.22% coverage with 544.74 kN and did not improve sharpness. These results quantify marginal predictive uncertainty and do not establish subgroup-conditional coverage or structural reliability.
6. For the three specimens with complete post-peak records, the two tested geogrid specimens exhibited higher normalized residual-load ratios and CAI values than the tested steel specimen. This case-specific observation is consistent with tensile bridging after punching, but the experiments provide complementary mechanics-consistency and post-peak evidence only; they do not constitute predictive or causal validation, establish general geogrid superiority, or support a design rule. Quantitative CAI evidence remains restricted to these three completely digitized records.
7. Source-grouped subgroup performance was R^2^ = 0.886 for interior, 0.309 for edge, and −0.851 for corner connections. The sparse edge/corner results require explicit applicability warnings and additional testing.

Overall, the framework is a research-oriented decision-support workflow, not a replacement for code design. Its output should be used only within the represented domain and together with design resistance, safety factors, detailing requirements, and engineering review.

## 6. Future Research

Future research should prioritize the expansion and diversification of the experimental evidence base, particularly through new and genuinely independent testing programs for edge and corner slab–column connections, which remain sparsely represented in the present database. Prospective validation using experimental campaigns or external datasets not represented in the current literature compilation is also required to assess transferability beyond the evidence base used in this study.

A second priority is the systematic replication of matched geogrid-reinforced post-peak experiments with complete load–displacement acquisition. Such testing would enable more rigorous assessment of residual capacity, deformation behavior, collapse-arrest characteristics, and post-failure integrity than is possible from the three complete post-peak records currently available.

As subgroup sample sizes increase, future work should investigate conditional or locally adaptive uncertainty-quantification methods, including subgroup-aware conformal approaches, rather than relying solely on marginal coverage. Formal structural-reliability analysis should be considered only after representative probabilistic distributions and dependence structures for the governing input variables have been established from appropriate experimental or field evidence.

Future studies should also extend mechanics-based benchmarking to additional formulations, including fib Model Code/CSCT-based approaches, where the required deformation, rotation, geometry, and material variables can be reconstructed consistently across specimens. Finally, the implemented research-screening GUI should undergo prospective validation, usability assessment, interface refinement, and evaluation of warning and uncertainty communication before consideration for broader engineering deployment.

Overall, further increases in algorithmic complexity are considered secondary to acquiring broader, more diverse, and genuinely independent experimental evidence, improving uncertainty characterization, and establishing prospective validation under conditions not represented in the current literature database.

## Figures and Tables

**Table 1:** Validation-centered methodological positioning of representative machine-learning studies on punching-shear assessment.

Study	Database/Structural Scope	Reported Model Evaluation	Source-Grouped Validation	Fully Source-Disjoint Testing	Configuration-Specific Transfer	XAI/Mechanics Interpretation	Uncertainty/Applicability	Engineering/Implementation Context
Nguyen et al. (2021) [12]	497 RC interior slab–column connections without shear reinforcement	Training/testing evaluation; XGBoost compared with ANN and RF	Not identified in the reported evaluation	Not identified in the reported evaluation	Interior connections only	Relative significance of input variables	Not identified as a dedicated component	Four design codes and three empirical models; GUI
Mangalathu et al. (2021) [34]	380 two-way RC flat slabs at interior supports without transverse reinforcement	Random 70/30 training-test split; eight regression approaches	Not identified in the reported evaluation	Not identified in the reported evaluation	Interior connections only	SHAP-based interpretation	Not identified as a dedicated component	Code provisions and empirical-model comparison
Liang et al. (2022) [17]	RC slab–column database addressing punching resistance and failure mode	Comparative ML modeling with XGBoost	Not identified in the reported evaluation	Not identified in the reported evaluation	Not identified as a dedicated transfer assessment	SHAP and PDP	No dedicated calibrated prediction-interval or applicability-domain framework identified	Mechanics-oriented interpretation of influential parameters
Shen et al. (2022) [35]	610 experimental RC slab–column records	ANN, DT, RF, and XGBoost surrogate models	Not identified in the reported evaluation	Not identified in the reported evaluation	Not identified as a dedicated transfer assessment	SHAP-based interpretation	Monte Carlo simulation and reliability-index assessment	Reliability-oriented assessment; empirical-model comparison
Yan and Xie (2024) [22]	337 RC flat slabs with transverse reinforcement	SVR, DT, RF, and XGBoost with Bayesian optimization	Not identified in the reported evaluation	Not identified in the reported evaluation	Not identified as a dedicated transfer assessment	SHAP	No dedicated calibrated prediction-interval or applicability-domain framework identified	Design provisions and CSCT comparison
Ren and Liu (2025) [36]	594 experimental RC slab–column tests without shear reinforcement	Probabilistic NGBoost compared with SVM, GP, RF, and XGBoost	Not identified in the reported evaluation	Not identified in the reported evaluation	Not identified as a dedicated transfer assessment	SHAP and Sobol sensitivity analysis	Probabilistic prediction and NGBoost-Monte Carlo reliability assessment	Reliability-oriented assessment
Shah et al. (2026) [37]	378 validated RC flat-slab tests after outlier screening	Eight ML algorithms with systematic optimization and comparative evaluation	Not identified in the reported evaluation	Not identified in the reported evaluation	Not identified as a dedicated transfer assessment	SHAP and PDP	No dedicated source-transfer uncertainty or applicability-domain framework identified	Engineering/mechanics-oriented interpretation
Present study	1068 specimens from 111 traceable literature-source groups; 983 interior, 51 edge, and 34 corner connections	Specimen-random five-fold benchmark: XGBoost R^2^ = 0.932	Yes; leakage-safe nested source-grouped validation: R^2^ = 0.858; globally tuned grouped-grid estimate: R^2^ = 0.881	Yes; held-out literature sources: R^2^ = 0.793	Yes; grouped R^2^ = 0.886 for interior, 0.309 for edge, and −0.851 for corner connections	SHAP + held-out grouped permutation importance + feature ablation; associative interpretation only	Source-disjoint conformal diagnostics + quantitative applicability-domain screening + bounded sensitivity analysis	ACI 318-19/EC2 nominal-error context; separate nine-specimen post-failure program; bounded research-screening GUI

**Table 2:** Descriptive statistics of the principal experimental variables included in the assembled database.

Variable	Unit	Minimum	Maximum	Mean	Median	Standard Deviation	COV (%)
Concrete compressive strength, (*f_c_*)	MPa	9.40	208.20	40.59	34.34	26.53	65.37
Effective slab depth, (d)	mm	13.80	500.00	101.31	100.00	46.76	46.15
Flexural reinforcement ratio, (*ρ_l_*)	%	0.00	15.60	1.08	1.00	0.87	79.88
Critical punching perimeter, (*u*_1_)	mm	279.92	3904.00	1114.73	1058.82	480.66	43.12
Experimental punching shear capacity, (*V*_exp_)	kN	3.59	2681.00	310.31	250.00	270.31	87.11

**Table 3:** Distribution of slab–column connection types included in the experimental dataset.

Connection Type	Number of Specimens	Percentage (%)
Interior	983	92.0
Edge	51	4.8
Corner	34	3.2
Total	1068	100.0

**Table 4:** Audited variables used in the validated 16-feature models and the experimental targets.

Symbol	Physical Meaning	Type	Unit	Inclusion and Reporting Basis
d	Effective depth; shear-transfer depth	Measured	mm	Complete; central punching-shear geometry
u1	Critical punching perimeter	Measured	mm	Complete; available resisting perimeter
fc	Concrete compressive strength	Measured	MPa	Complete; concrete resistance
fy	Longitudinal-steel yield strength	Measured	MPa	Complete; reinforcement capacity
ρl	Longitudinal reinforcement ratio	Measured	% points	Complete; crack control and dowel action; 1.00 means 1.00%
Vf	Fiber volume fraction	Measured	%	Complete after QC; crack-bridging descriptor
Ac	Loaded column area	Measured	mm^2^	Complete; load-transfer geometry
e/d	Normalized eccentricity	Derived from reported e and d	–	Complete; dimensionless loading eccentricity
Type indicators	Interior, edge, corner one-hot indicators	Categorical encoding	0/1	Complete; connection boundary configuration
√fc	Concrete-strength transformation	Engineered	MPa^0.5^	Complete; prespecified strength scaling
Ac/d^2^	Normalized loaded area	Engineered	–	Complete; relative column geometry
ρlfy	Reinforcement-capacity interaction	Engineered	%·MPa	Complete; prespecified reinforcement descriptor
ρl√fc	Reinforcement–concrete interaction	Engineered	%·MPa^0.5^	Complete; prespecified coupled descriptor
u1d	Perimeter–depth interaction	Engineered	mm^2^	Complete; effective shear-transfer region
Vu	Experimental punching capacity	Target	kN	Complete measured response

**Table 5:** Outcome of the feature-audit procedure.

Feature-Audit Category	Variables	Decision and Reason
Retained direct predictors	*d*, *u*1, *fc*, *fy*, *ρl*, *Vf*, *Ac*	Seven reported engineering variables used in the validated model.
Retained loading and configuration	*e*/*d*; Type_Interior; Type_Edge; Type_Corner	Dimensionless eccentricity and three one-hot connection indicators used in the validated model.
Retained engineered descriptors	√*fc*; *Ac*/*d*2; *ρlfy*; *ρl*√*fc*; *u*1*d*	Five prespecified mechanics-informed descriptors generated only from predictors.
Traceability only	*c*1; *c*2	Source-level geometry fields; not used in the validated 16-feature model.
Excluded from validated model	*h*	Missing in 946 of 1068 records; retained only as a source-level traceability field.
Excluded	*m*	Unstable definition, unit, and reproducible provenance across source studies.

**Table 6:** Documented hyperparameter status of the investigated machine-learning models.

Algorithm	Hyperparameter	Search Values
XGBoost	n_estimators	300, 500, 800
XGBoost	max_depth	3, 4, 5, 6
XGBoost	learning_rate	0.03, 0.05, 0.10
XGBoost	subsample	0.80, 0.90, 1.00
XGBoost	colsample_bytree	0.80, 0.90, 1.00
XGBoost	min_child_weight	1, 3, 5
XGBoost	gamma	0.00, 0.05, 0.10
XGBoost	reg_alpha	0.00, 0.01
XGBoost	reg_lambda	1.0, 3.0
Random Forest	n_estimators	300, 500, 800
Random Forest	max_depth	None, 10, 20, 30
Random Forest	min_samples_split	2, 5, 10
Random Forest	min_samples_leaf	1, 2, 4
Random Forest	max_features	sqrt, 0.7, 1.0
CatBoost	iterations	300, 500, 800
CatBoost	depth	4, 6, 8
CatBoost	learning_rate	0.03, 0.05, 0.10
CatBoost	l2_leaf_reg	1, 3, 5, 7

**Table 7:** Reproducibility status and retained hyperparameter settings of the investigated machine-learning models.

Model	Required Final Setting	Status in Supplied Package
Random Forest	Selected n_estimators, max_depth, min_samples_split, min_samples_leaf, max_features; random_state = 42	Legacy specimen-random workflow; selected values were not archived in the supplied reproducibility package
XGBoost	Selected n_estimators, max_depth, learning_rate, subsample, colsample_bytree, min_child_weight, gamma, reg_alpha, reg_lambda; random_state = 42	Legacy workflow as in Table 6; confirmatory grouped grid: 432 candidates, GroupKFold (5), R^2^; selected 800 trees, depth 3, learning rate 0.03, subsample 0.8, colsample 1.0, min_child_weight 1, reg_lambda 1, random_state 42
CatBoost	Selected iterations, depth, learning_rate, l2_leaf_reg; random_seed = 42	Legacy specimen-random workflow; selected values were not archived in the supplied reproducibility package

**Table 8:** Five-fold specimen-level cross-validation performance of the investigated machine-learning models.

Model	Mean	Std.	Mean MAE (kN)	Mean RMSE (kN)
XGBoost	0.927	0.015	43.57	72.57
RF	0.921	0.014	44.24	75.55
CatBoost	0.911	0.034	46.28	78.79

**Table 9:** Bias-related statistical indicators of the optimized XGBoost model for the withheld specimen-level test dataset.

Metric	Value
MBE (kN)	12.01
MPE (%)	−3.90
MAPE (%)	17.39

**Table 10:** Comparison of specimen-level, source-grouped, and source-disjoint XGBoost validation protocols.

Validation Strategy	R^2^	MAE (kN)	RMSE (kN)	MAPE (%)	Interpretation
Specimen-random 5-fold benchmark	0.932	42.68	70.54	18.09	Within-database specimen-level performance
Source-grouped 5-fold with globally selected grouped-grid hyperparameters	0.881	60.71	93.22	29.84	Tuned grouped estimate; hyperparameters selected globally using grouped CV
Nested source-grouped GroupKFold (leakage-safe)	0.858	66.38	101.80	33.07	More conservative source-grouped estimate; hyperparameter selection nested within each outer fold
Source-disjoint 80/20	0.793	55.38	83.73	23.05	Held-out literature sources

**Table 11:** Comparative predictive performance of the proposed XGBoost model and current design provisions using the withheld specimen-level test dataset.

Method	R^2^	MAE	RMSE	MAPE	Mean (*V*_pred_/*V_exp_*)
ACI 318-19	0.762	106.26	137.12	47.70	0.936
Eurocode 2	0.322	172.60	231.71	55.67	0.484
Proposed XGBoost	0.924	44.10	77.71	17.39	1.039

**Table 12:** Statistical summary of the perturbation-based robustness analysis.

Scenario	Mean Capacity (kN)	Standard Deviation (kN)	Coefficient of Variation (%)	5th Percentile (kN)	95th Percentile (kN)
Interior/Concentric	308.20	23.15	7.51	257.40	334.37
Edge–Corner/Eccentric	272.57	77.38	28.39	203.39	386.04

**Table 13:** Post-peak metrics for the three specimens with complete digitized load–displacement records.

Specimen	Peak Load (kN)	Final Load (kN)	CAI	Residual-Load Ratio	Final Deflection (mm)
Geogrid-1	17.1	9.2	0.640	0.538	15.57
Geogrid-2	22.2	12.9	0.807	0.581	20.87
Steel-S-5-3	36.0	12.0	0.560	0.333	16.04

**Table 14:** Comparison of grouped GridSearch and nested Optuna/TPE results. The protocols differ in nesting and do not isolate optimizer superiority.

Method	Selection Protocol	R^2^	MAE (kN)	RMSE (kN)	MAPE (%)
Grouped GridSearch	Global GroupKFold selection	0.881	60.71	93.22	29.84
Optuna/TPE	Nested GroupKFold (5 × 5; 300 trials/fold)	0.858	66.38	101.80	33.07

## Data Availability

The original contributions presented in this study are included in the article/Supplementary Materials. Further inquiries can be directed at the corresponding author.

## Supplementary Materials

The following supporting information can be downloaded at https://www.mdpi.com/article/10.3390/ma19204351/s1. The supporting information accompanying this article includes the quality-controlled dataset, source-grouped validation assignments and leakage audits, out-of-fold predictions, uncertainty and applicability-domain analyses, interpretability outputs, optimization and sensitivity analyses, reproducibility scripts and computational-environment information, and the research-screening GUI package.

## Abbreviations

The following abbreviations are used in this manuscript:

ACI	American Concrete Institute
AI	Artificial intelligence
ANN	Artificial neural network
CAI	Collapse-Arrest Index
CDF	Cumulative distribution function
COV	Coefficient of variation
CSCT	Critical Shear Crack Theory
CV	Cross-validation
DT	Decision tree
EC2	Eurocode 2
FRP	Fiber-reinforced polymer
GFRP	Glass-fiber-reinforced polymer
GP	Gaussian process
KDE	Kernel density estimation
MAE	Mean absolute error
MAPE	Mean absolute percentage error
MBE	Mean bias error
MCS	Monte Carlo simulation
ML	Machine learning
MPE	Mean percentage error
MPIW	Mean prediction interval width
NGBoost	Natural Gradient Boosting
PDF	Probability density function
PDP	Partial dependence plot
RC	Reinforced concrete
RF	Random Forest
RMSE	Root mean square error
SHAP	SHapley Additive exPlanations
SVM	Support vector machine
SVR	Support vector regression
UHPFRC	Ultra-high-performance fiber-reinforced concrete
XAI	Explainable artificial intelligence
XGBoost	Extreme Gradient Boosting
